# A plasmid-encoded inactive toxin–antitoxin system MtvT/MtvA regulates plasmid conjugative transfer and bacterial virulence in *Pseudomonas aeruginosa*

**DOI:** 10.1093/nar/gkaf075

**Published:** 2025-02-14

**Authors:** Meng Li, Hua Guo, Lecheng Wang, Ruixue Tao, Gaoyu Song, Linke Cao, Wenbo Yan, Ziyuan Wu, Qian Liu, Yaodong Chen, Lei Gong, Tietao Wang, Yani Zhang

**Affiliations:** Key Laboratory of Resource Biology and Biotechnology in Western China, Ministry of Education, Provincial Key Laboratory of Biotechnology, College of Life Sciences, Northwest University, Xi’an, Shaanxi 710069, People’s Republic of China; Science and Education Department, Xi’an Fifth Hospital, Xi’an, Shaanxi 710082, People’s Republic of China; Science and Education Department, Xi’an Fifth Hospital, Xi’an, Shaanxi 710082, People’s Republic of China; Key Laboratory of Resource Biology and Biotechnology in Western China, Ministry of Education, Provincial Key Laboratory of Biotechnology, College of Life Sciences, Northwest University, Xi’an, Shaanxi 710069, People’s Republic of China; Key Laboratory of Resource Biology and Biotechnology in Western China, Ministry of Education, Provincial Key Laboratory of Biotechnology, College of Life Sciences, Northwest University, Xi’an, Shaanxi 710069, People’s Republic of China; Key Laboratory of Resource Biology and Biotechnology in Western China, Ministry of Education, Provincial Key Laboratory of Biotechnology, College of Life Sciences, Northwest University, Xi’an, Shaanxi 710069, People’s Republic of China; Key Laboratory of Resource Biology and Biotechnology in Western China, Ministry of Education, Provincial Key Laboratory of Biotechnology, College of Life Sciences, Northwest University, Xi’an, Shaanxi 710069, People’s Republic of China; Key Laboratory of Resource Biology and Biotechnology in Western China, Ministry of Education, Provincial Key Laboratory of Biotechnology, College of Life Sciences, Northwest University, Xi’an, Shaanxi 710069, People’s Republic of China; Key Laboratory of Resource Biology and Biotechnology in Western China, Ministry of Education, Provincial Key Laboratory of Biotechnology, College of Life Sciences, Northwest University, Xi’an, Shaanxi 710069, People’s Republic of China; Key Laboratory of Resource Biology and Biotechnology in Western China, Ministry of Education, Provincial Key Laboratory of Biotechnology, College of Life Sciences, Northwest University, Xi’an, Shaanxi 710069, People’s Republic of China; Key Laboratory of Resource Biology and Biotechnology in Western China, Ministry of Education, Provincial Key Laboratory of Biotechnology, College of Life Sciences, Northwest University, Xi’an, Shaanxi 710069, People’s Republic of China; Science and Education Department, Xi’an Fifth Hospital, Xi’an, Shaanxi 710082, People’s Republic of China; Key Laboratory of Resource Biology and Biotechnology in Western China, Ministry of Education, Provincial Key Laboratory of Biotechnology, College of Life Sciences, Northwest University, Xi’an, Shaanxi 710069, People’s Republic of China; Key Laboratory of Resource Biology and Biotechnology in Western China, Ministry of Education, Provincial Key Laboratory of Biotechnology, College of Life Sciences, Northwest University, Xi’an, Shaanxi 710069, People’s Republic of China

## Abstract

Plasmid-encoded toxin–antitoxin (TA) systems are known for their role in plasmid maintenance via post-segregational killing. Here, we identified an inactive type II TA system, MtvT/MtvA (MtvTA), encoded on the conjugative plasmid pPAD8 from the clinical *Pseudomonas aeruginosa* strain PAD8. Despite its annotation as a toxin, MtvT exhibited no detectable toxicity in our assays. Interestingly, the deletion of the MtvTA significantly increased the transfer efficiency of pPAD8 from PAD8 to *P. aeruginosa* strain PAO1. Functional assays revealed that the MtvTA complex negatively regulates plasmid transfer by binding to the promoters of *dot*/*icm* system genes. In addition, pPAD8^Δ^*^mtvTA^* attenuated the pathogenicity of the host strain compared to pPAD8, highlighting a regulatory role for MtvTA in virulence. Mechanistically, the MtvTA complex positively regulates the type III and type VI secretion systems and pyocyanin biosynthesis by directly binding to the promoters of *exsA* and *rsmY*/*rsmZ* and indirectly influencing *lasI* expression, respectively. These findings provide new insights into the regulatory roles of an inactive plasmid-encoded TA system, expanding our understanding of the interplay between plasmids and their bacterial hosts.

## Graphical Abstract

**Graphical Abstract:**
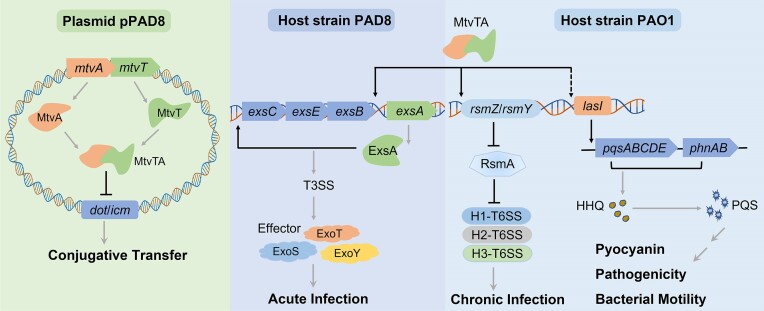

## Introduction

In recent years, clinical strains harboring large plasmids have been increasingly identified [1, 2]. These plasmids carry a wide variety of genetic elements, such as resistance determinants, virulence genes, and other adaptive factors essential for the environmental adaptability of the host strain [3]. Plasmids that can transfer between bacterial cells are known as conjugative plasmids [4]. The spread of genetic elements facilitated by large conjugative plasmid via horizontal gene transfer (HGT) among different strains and species plays a key role in bacterial evolution and poses an escalating threat to the treatment of bacterial infections [3, 5, 6]. However, the mechanisms underlying plasmid conjugative transfer and their impact on host strain pathogenicity remain poorly understood.

Conjugative transfer mediated by the type IV secretion system (T4SS) is a well-known mechanism for HGT among bacteria [7]. In Gram-negative bacteria, T4SS are classified into two subtypes, T4ASS and T4BSS, represented by the *Agrobacterium tumefaciens* VirB/VirD4 system and *Legionella pneumophila* Dot/Icm system, respectively [8–10]. T4SS functions as the conjugation system (facilitating DNA transfer) and effector translocation system (delivering effector macromolecules) [7, 11, 12]. Additionally, for conjugative plasmids, the success of vertical transmission is crucial for their maintenance in bacterial communities [13]. Toxin–antitoxin (TA) systems encoded by plasmid are widely known for stabilizing plasmids via a mechanism called “post-segregational killing” (PSK) or “plasmid addiction” [14], which ensures plasmid stability within the cell by killing daughter cells that fail to inherit the plasmid during cell division.

TA systems, consisting of a pair of genes encoding a stable toxin and an unstable antitoxin, are widely distributed in bacterial genomes, particularly on conjugative plasmids [15]. Currently, eight types of TA systems (types I–VIII) are classified based on the nature of antitoxin and the associated mechanism of neutralizing the toxicity [15]. Type II TA systems are the most prevalent and have been extensively studied within the large family of TA systems [14, 16]. In a typical type II system, both TA genes are arranged in an operon, with the antitoxin proteins blocking the toxicity of the toxin through directly protein–protein interaction [14, 17]. Most type II antitoxins function as dual-domain proteins, with an N-terminal DNA-binding domain and a C-terminal toxin-binding domain [18]. TA systems were originally discovered for stabilizing low copy number plasmids via PSK [19]. Studies have revealed that antitoxins can also bind to the promoter regions of other genes to regulate their expression [20–24]. For instance, in *Escherichia coli*, the antitoxin MqsA regulates the stress response and biofilm formation by binding to the promoters of *rpoS* and *csgD*, respectively [20, 21]. Similarly, the type II antitoxin HigA in *Pseudomonas aeruginosa* regulates virulence by binding to the promoters of the *mvfR*, *amrZ*, and *exsA* [22, 24]. These observations suggest that TA systems play a significant role in bacterial pathogenicity.

*Pseudomonas aeruginosa* is a Gram-negative opportunistic pathogen causing devastating chronic and acute infections in individuals with cystic fibrosis and those who are immunocompromised [25]. It employs numerous strategies to establish persistent infections in the host, including quorum sensing (QS) system, type III secretion system (T3SS), type VI secretion system (T6SS), and pyocyanin production (an endemic pigment of *P. aeruginosa*) [26–28]. To date, at least four QS pathways, including *las*, *rhl*, *pqs*, and *iqs*, have been characterized in *P. aeruginosa* [29]. The QS system plays a pivotal role in regulating antibiotic resistance, motility behavior, biofilm formation, and pyocyanin production [25]. Secretion systems in *P. aeruginosa*, such as T3SS and T6SS, can directly inject effectors into surrounding cells, triggering acute infection and chronic infection, respectively [26]. T3SS gene expression is directly regulated by the master regulator ExsA [30], while T6SS genes are regulated by the transcriptional regulator AmrZ and the post-transcriptional regulator RsmA [31]. The soluble RNA repressors RsmY and RsmZ, which are directly controlled by GacS/GacA, negatively regulate the activity of RsmA [32]. RsmA promotes T3SS gene expression while inhibits T6SS gene expression [31, 33].

Here, we identified an inactive type II TA system, designated MtvT/MtvA (MtvTA), that modulates plasmid transfer and bacterial virulence. It is encoded on the conjugative plasmid pPAD8 from the clinical *P. aeruginosa* strain PAD8. We found that overexpression of the putative toxin MtvT in both *P. aeruginosa* and *E. coli* DH5α did not inhibit bacterial growth. Interestingly, deletion of the *mtvTA* operon resulted in a significant increase in the transfer frequency of pPAD8 from PAD8 to *P. aeruginosa* strain PAO1. We discovered that the MtvTA complex directly binds to the promoter regions of *dotA*,*dotD*,*icmP*, and *icmL* of the *dot*/*icm* system, inhibiting the conjugative transfer of pPAD8, while binding to the promoters of *exsA* and *rsmY*/*rsmZ* upregulates the T3SS and T6SS systems. Furthermore, MtvTA regulates pyocyanin biosynthesis by indirectly regulating *lasI* expression. Thus, the MtvTA system from plasmid pPAD8 exhibits novel functions in plasmid transfer and host bacterial physiology. Our findings provide novel insights into the regulatory roles of an inactive plasmid-encoded TA system, expanding our understanding of the interplay between plasmids and their bacterial hosts.

## Materials and methods

### Bacterial strains and growth conditions

The bacterial strains and plasmids used in this study are listed in Supplementary Table S1. All bacterial strains were cultured in LB medium or *Pseudomonas* isolation agar (PIA) at 37°C. When needed, antibiotics were supplemented to the medium at the following final concentrations: 50 μg/ml gentamicin, 100 μg/ml tetracycline, 300 μg/ml carbenicillin, and 300 μg/ml fosfomycin for *P. aeruginosa*; 50 μg/ml kanamycin, 10 μg/ml tetracycline, and 100 μg/ml carbenicillin for *E. coli*.

### Construction of plasmids

Plasmid construction was performed using standard molecular biology techniques. All primers used in this study are listed in Supplementary Table S2. For example, to construct plasmids pBBR1-*mtvA*, pBBR1-*mtvT*, and pBBR1-*mtvTA*, the DNA fragments were amplified by PCR using the corresponding primer pairs pBBR1-*mtvA*-F/R, pBBR1-*mtvT*-F/R, and pBBR1-*mtvA*-F/pBBR1-*mtvT*-R, respectively. The purified PCR products were digested and cloned into pBBR1MCS-5 plasmid. For construction of pMMB67EH‐*mtvTA*-Flag plasmid, the PCR products were amplified using the primer pairs pMM‐*mtvTA*-F/R and the digested PCR products were subsequently cloned into the pMMB67EH-Flag plasmid.

### Construction of deletion mutants

For the construction of gene knockout mutants, a *sacB*-based strategy was employed as described previously [34]. To construct the *mtvTA* deletion mutant, ∼1000 bp of homologous sequences upstream and downstream of the *mtvTA* operon were directly amplified from PAD8 using primer pairs pEX-*mtvTA*-up-F/R and pEX-*mtvTA*-down-F/R. The two PCR products were digested and then cloned into digested vector pEX18Ap, yielding pEX18Ap*-mtvTA*. A 0.9-kb gentamicin resistance cassette was cloned into pEX18Ap-*mtvTA*, yielding pEX18Ap-*mtvTA*-Gm. The resultant plasmids were electroporated into PAD8 with selection for gentamicin resistance. The isolated colonies were streaked on PIA plates containing 50 μg/ml gentamicin and 10% sucrose, which selects for a double-crossover event and gene replacement. The resulting mutant was named PAD8(pPAD8^Δ^*^mtvTA^*). A similar strategy was used to construct the pPAD8^Δ^*^mtvA^*, pPAD8^Δ^*^mtvT^*, pPAD8^Δ^*^dinJ^*^/^*^yafQ^*, and Δ*glpT* deletion mutants. All mutants were verified by PCR and DNA sequencing.

### Protein expression and purification

The his-tagged protein was expressed in *E. coli* strain Transetta (DE3). Bacteria cultures were cultivated at 37°C in LB medium to an OD_600_ of 0.6, then shifted to 16°C and induced with 0.5 mM Isopropyl-β-D-1-thiogalactopyranoside (IPTG) for an additional 20 h. The harvested cells were resuspended in His buffer A (10 mM Tris–HCl, 500 mM NaCl, pH 8.0) and disrupted by sonication. The target proteins were purified using a 5-ml HisTrap HP column (GE Healthcare, USA) with His buffer B (10 mM Tris–HCl, 500 mM NaCl, 500 mM imidazole, pH 8.0). For the purification of GST-tagged proteins, the expression plasmid pGEX6p-1 was used. The target protein was purified using a 5-ml GSTrap HP column (GE Healthcare) with GST buffer A (50 mM Tris–HCl, 150 mM NaCl, pH 8.0) and GST buffer B (50 mM Tris–HCl, 150 mM NaCl, 10 mM glutathione, pH 8.0) according to the manufacturer’s instructions. The proteins were concentrated and stored at −80°C until used.

### GST pull‐down assay

To verify the interaction between MtvA and MtvT *in vitro*, a GST pull-down assay was performed as described previously [35]. Briefly, 100 μg of purified GST and an equal amount of GST‐MtvA were mixed with 30 μl of pre-washed glutathione magnetic beads (Promega, Madison, WI, USA) at 4°C for 2 h. The unbound proteins were washed away with GST buffer A. Then, 20 μg of His-MtvT was added to the pre‐blocked beads and incubated at 4°C for 4 h. The beads were then washed five times with GST buffer A. The retained proteins were separated by sodium dodecyl sulfate–polyacrylamide gel electrophoresis (SDS–PAGE), followed by western blot using standard protocol with GST (Immunoway) and His (TransGen Biotech) antibodies. The signal was detected using an ECL Plus Kit (GE Healthcare, Piscataway, NJ, USA).

### Electrophoretic mobility shift assay

Proteins were mixed with DNA probes in 20 μl of the gel shift-loading buffer (20 mM HEPES, 100 mM NaCl, 1 mM DTT, 3 μg/ml sheared salmon sperm DNA, pH 8.0). After incubation for 25 min at room temperature, the samples were analyzed by 8% polyacrylamide gel electrophoresis in 0.5×TBE buffer. The gels were stained for 10 min with GelRed staining solution (TransGen Biotech) and analyzed using a Tanon 5200 image analysis system (Tanon Technologies).

### Bacterial two-hybrid assay

The interaction between MtvA and MtvT was examined using a bacterial two-hybrid (BACTH) system as described previously [18]. The coding regions of *mtvA* and *mtvT* were cloned into the pKT25 or pUT18C plasmids, respectively, and co-transformed into *E. coli* BTH101 competent cells. Co-transformed cells were plated onto LB agar plates supplemented with kanamycin (50 μg/ml), ampicillin (100 μg/ml), X-gal (20 μg/ml), and IPTG (0.5 mM) and cultivated at 30°C for 24 h. The cells harboring pKT25-*zip*/pUT18C-*zip* were used as positive controls, while the cells harboring pKT25/pUT18C-*mtvA* and pKT25-*mtvT*/pUT18C were used as negative controls.

### Measurement of the promoter activities by *lux* fusions

The plasmid pMS402 carrying a promoterless *luxCDABE* reporter gene cluster was used to construct promoter–*luxCDABE* reporter fusions as previously described [36]. The *mtvTA* promoter region was amplified by PCR and cloned into the pMS402 to yield *mtvTA*-*lux*. Other promoter–*luxCDABE* reporter plasmids were constructed using the same strategy. The resulting plasmids were verified by DNA sequencing.

Expression of the *lux*-based reporters was measured as counts per second of light production as previously described [37]. Briefly, overnight cultures of the reporter strains were diluted to OD_600_ of 0.2 with fresh LB medium and cultured for another 2 h. Subsequently, the cultures were diluted 1:20 with fresh LB and transferred into a black 96-well plate with a transparent bottom. A total of 50 μl of sterilized mineral oil was added to the culture. Promoter activities and bacterial growth (OD_600_) were continuously measured every 30 min for 24 h using a Synergy 2 Plate Reader (BioTek).

### Conjugation experiments

Conjugation experiments were performed as previously described with slight modifications [38]. The fosfomycin-resistant *P. aeruginosa* PAO1 strain (Δ*glpT*), generated by deleting the *glpT* gene encoding the fosfomycin transporter, was used as the recipient strain. The PAD8(pPAD8) strain carrying gentamicin resistance (pPAD8::*armA*), generated by inserting *armA* into a pseudogene (gene ID: IBG29_30210) of plasmid pPAD8 using homologous recombination, was used as the donor strain. Donor and recipient strains were grown overnight in LB broth with the appropriate antibiotics at 37°C, and then diluted 1:100 into fresh LB and incubated for 4 h, respectively. For solid mating, the donor and recipient strains were mixed in a 4:1 ratio in 1 ml LB broth and co-cultured on solid LB agar, which were incubated at 37°C for 12 h. For liquid mating, the donor and recipient strains were mixed in a 4:1 ratio in 1 ml LB broth, and the suspension was incubated at 37°C for 12 h without shaking. After 12 h incubation, the mating mixtures were resuspended in LB broth, serially diluted, and spread onto LB agar supplemented with the appropriate antibiotics selective for donors (300 μg/ml fosfomycin) and transconjugants (50 μg/ml gentamicin and 300 μg/ml fosfomycin). Conjugative transfer frequencies were calculated as the ratio of transconjugants to donors.

### RNA extraction and quantitative real-time PCR

Overnight bacterial cultures were diluted 1:100 with fresh LB broth and subcultured to an OD_600_ of 0.6. Then, total RNA was isolated from the bacteria using the RNAprep pure cell/bacteria kit (Tiangen Biotech, China). Complementary DNA (cDNA) was synthesized using PrimeScript Reverse Transcriptase (TaKaRa, China) with random primers. Quantitative RT-PCR (qRT-PCR) was performed using SYBR Green PCR master mix (Abm, Canada) on the CFX96 Real-Time PCR Detection System (Bio-Rad Laboratories, USA). The 30S ribosomal protein gene *rpsL* was used as an internal control.

### RNA-seq and data analysis

The PAD8(pPAD8), PAO1(pPAD8), PAD8(pPAD8^Δ^*^mtvTA^*), and PAO1(pPAD8^Δ^*^mtvTA^*) strains were grown in LB medium at 37°C until OD_600_ reached ∼0.8. Total RNA was extracted immediately upon cell harvest using the TruSeq™ Stranded Total RNA Library Prep Kit according to the manufacturer’s instructions. The cDNA library was created by reverse transcription, followed by ends preparation, adaptor ligation, and PCR amplification step by step from the RNA samples. The cDNA libraries were then sequenced on an Illumina NovaSeq 6000 platform (Majorbio-Shanghai). Each RNA-seq sample assay was repeated three times. RNA-seq reads were mapped to the reference genomes of *P. aeruginosa* PAD8 (NZ_CP061073.2) and *P. aeruginosa* PAO1 (NC_002516.2), as provided by National Center for Biotechnology Information, using Bowtie 2. Only uniquely mapped reads were kept for the subsequent analyses. Differentially expressed genes (DEGs) were identified using DESeq2 (Benjamini–Hochberg adjusted *P*< .05 and |log_2_fold change| > 1) for the comparisons between PAD8(pPAD8) and PAD8(pPAD8^Δ^*^mtvTA^*) as well as PAO1(pPAD8) and PAO1(pPAD8^Δ^*^mtvTA^*). The identified DEGs were enriched by Kyoto Encyclopedia of Genes and Genomes (KEGG) pathway. These data have been uploaded under BioProject accession numbers PRJNA1127266 and PRJNA1127263, respectively.

### Biofilm formation assay

Biofilm formation was assessed as described previously [35]. Briefly, overnight cultures grown in LB medium were diluted (1:1000) into 1 ml of fresh LB medium in glass tube and incubated statically at 25°C for 22 h. Biofilms attached to the sides of the glass tubes were gently washed twice with sterile water and stained with crystal violet (CV). The biofilm biomass was quantified by dissolving the CV‐stained biofilm in 95% ethanol, and the absorbance of the CV solution was measured at 590 nm.

### Measurement of pyocyanin production

Pyocyanin was extracted from culture supernatants and quantified as previously described [36]. Briefly, 5 ml of culture supernatant was mixed with 3 ml of chloroform and vortexed. The chloroform layer was transferred to a fresh tube and mixed with 1 ml of 0.2 M HCl. After centrifugation, the top layer was removed. Pyocyanin production was determined by measuring the absorbance of the resulting acidic pink layer at 520 nm. The concentration of pyocyanin (μg/ml) was calculated by multiplying the absorbance by a factor of 17.072.

### PQS production assay

*Pseudomonas* quinolone signal (PQS) extraction and analysis were performed as described previously [39]. Briefly, overnight bacterial cultures were diluted 100-fold in fresh LB medium and incubated at 37°C for 24 h. After incubation, 500 μl of the culture was mixed with 1 ml of acidified ethyl acetate, vortexed vigorously for 2 min, and centrifuged at 16 000 × *g* for 10 min. The upper organic layer was transferred to a fresh tube and allowed to dry overnight at room temperature. The following day, the dried extracts were dissolved in 50 μl of a solution containing acidified ethyl acetate and acetonitrile (1:1, v/v). The samples were then analyzed by thin-layer chromatography (TLC) and quantitated by densitometry using ImageJ software.

### Bacterial motility assay

Motility assays were performed as described previously with minor modifications [40]. The swarming medium contained 0.8% nutrient broth, 0.5% glucose, and 0.5% agar. The swimming medium consisted of 1% tryptone, 0.5% NaCl, and 0.3% agar. The twitching medium was LB broth supplemented with 1% agar. *Pseudomonas aeruginosa*cultures were diluted to an OD_600_ of 1.0, and 2 μl of the diluted culture was center spotted onto the surface of the agar plates. After the bacterial liquid was absorbed, the swimming plates were incubated at 30°C for 16 h, the twitching plates were incubated at 37°C for 24 h, and the swarming plates were incubated at 37°C for 16 h. Images were captured using the Tanon 2500 imaging system.

### *Galleria mellonella* killing assays

The *G. mellonella* infection model is a widely accepted animal model, and the experiments were performed as described previously [41]. *Pseudomonas aeruginosa* cells were grown in LB broth to an optical density of 1.0 at OD_600_, washed three times with sterile phosphate buffer saline (PBS), and then diluted in PBS to a final concentration of 10^4^ CFU/ml. A 100-μl Hamilton syringe was used to inject 20 μl of bacterial suspension into the last left proleg of *G. mellonella* via the last left proleg. The control group was injected with 20 μl of sterile PBS. Infected larvae were incubated and monitored in the dark at 37°C in an incubator. The number of dead caterpillars was recorded at each time point. Caterpillars were considered dead when they showed no movement in response to touch.

### Western blot analysis

Protein samples were separated by electrophoresis on 15% or 20% SDS–PAGE and transferred to polyvinylidene difluoride membranes. After blocking with 5% (w/v) skim milk in TBST buffer (50 mM Tris, 150 mM NaCl, 0.05% Tween 20, pH 7.4), the membranes were incubated with the appropriate primary antibodies: anti‐Flag (Sigma, Germany), anti‐GST (Zhongshan Golden Bridge Biotechnology, Beijing, China), and anti‐RNA polymerase α (Biolegend, California, USA). After three times washes with TBST buffer, the membranes were incubated with horseradish peroxidase‐conjugated rabbit (TransGen, Beijing, China, for GST) or mouse (TransGen, for Flag and RNAP) antibodies. Protein signals were detected using the ECL kit following the manufacturer’s protocol.

### Bioinformatics analysis

Plasmid pPAD8 was annotated using the NCBI Prokaryotic Genome Annotation Pipeline (PGAP, version 6.5) and the RAST server (version 2.0). Resistance genes were identified with ResFinder (version 4.3.3) and the RGI software from the CARD database. To identify pPAD8-like plasmids, BLASTn searches were conducted against the nonredundant GenBank database (up to June 2024) with pPAD8 as the query sequence. Molecular docking simulations were performed using AutoDock 4.2.6, with the MtvA protein structure generated by AlphaFold2 [42]. The palindromic sequence 5′-GGTAATAACATTGTTACTACC-3′ was used as the ligand. The 3D structure of the ligand–receptor complex was visualized using PyMOL.

## Results

### Identification of the conjugative plasmid pPAD8 in *P. aeruginosa* strain PAD8

We isolated the multidrug-resistant *P. aeruginosa* strain PAD8 from clinical environment (antimicrobial susceptibility results are summarized in Supplementary Table S3). The genomic DNA (gDNA) of PAD8 was sequenced using a hybrid approach, combining Illumina (short-read) and Nanopore (long-read) sequencing technologies. The final genome assembly revealed that PAD8 harbors a large circular plasmid, designated pPAD8 (GenBank accession no. NZ_CP061074.2), which is 140 400 bp in size and encodes 156 proteins. This plasmid contains genes involved in plasmid replication initiator (RepB), plasmid partitioning (ParA/ParB), conjugation transfer (*dot*/*icm* type IVB secretion system and *pil* operon), and the type IV CRISPR–Cas system (Supplementary Fig. S1A). Notably, pPAD8 does not carry any known antibiotic resistance genes.

**Figure 1.**
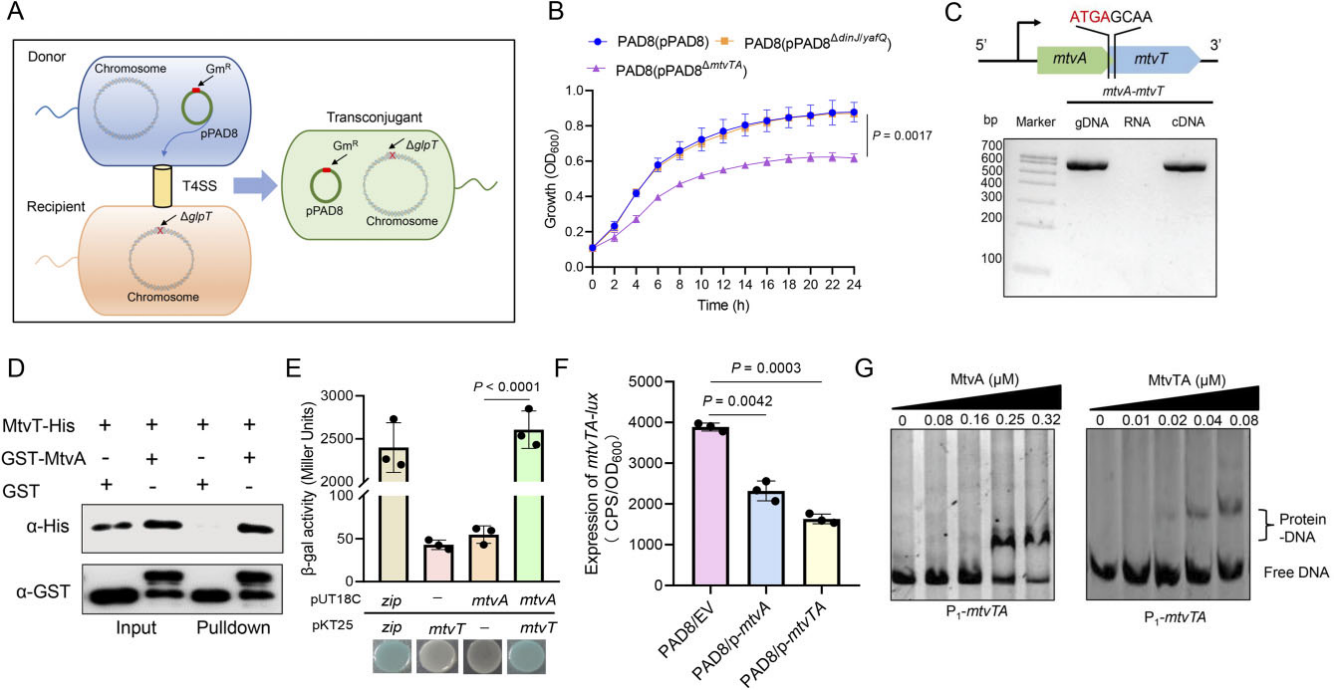
An inactive type II TA system MtvTA encoded on the conjugative plasmid pPAD8. (**A**) Schematic diagram of conjugative transfer of pPAD8. Donor: strain PAD8 with gentamicin resistance; recipient: strain PAO1Δ*glpT* with fosfomycin resistance; transconjugant: strain PAO1Δ*glpT*(pPAD8). The chromosome and plasmid pPAD8 are shown by a large circle and a small circle, respectively. The gentamicin resistance gene *armA* is indicated by a square. The deletion *glpT* gene is indicated by a “x”. T4SS: type IV secretion system. (**B**) Growth of the wild-type PAD8(pPAD8), the mutant PAD8(pPAD8^Δ^*^mtvTA^*), and the mutant PAD8(pPAD8^Δ^*^dinJ^*^/^*^yafQ^*) was determined using a Synergy 2 Microplate Reader (BioTek). Three independent cultures of each strain were tested, and error bars indicate the standard error of the mean (*n* = 3). (**C**) Co-transcription of *mtvA* and *mtvT*. The overlapped bases are ATGA. Primers were designed to amplify the whole coding region of *mtvA* and *mtvT*. Approximately 200 ng of cDNA reverse transcribed from the PAD8 RNA was used as the template; the same amount of PAD8 gDNA and RNA was used as the positive and negative controls, respectively. (**D**) GST pull‐down assays showing that MtvA interacts with MtvT. Purified MtvT was incubated with GST and GST-MtvA individually, and protein complexes were captured by glutathione beads. (**E**) The qualitative and quantitative analyses of the BACTH assay. BACTH assay and β-galactosidase assay were performed to assess the interactions between MtvA and MtvT. The strains expressing the pKT25‐*zip*/pUT18C‐*zip* plasmids were used as positive controls, and the strains expressing the pKT25-*mtvT*/pUT18C or pKT25/pUT18C‐*mtvA* plasmids were used as negative controls. (**F**) The promoter activity of *mtvTA* was measured by overexpressing *mtvA* or *mtvTA*. EV represents the empty vector pBBR1MCS-5. OD_600_, optical density at 600 nm. CPS, counts per second. (**G**) Electrophoretic mobility shift assay (EMSA) showing that MtvA and the MtvTA complex bind and shift P_1_-*mtvTA*. Each reaction mixture contains 1.0 ng/μl of PCR products of P1-mtvTA. Protein concentrations are indicated above the lane. For panels (E) and (F), the quantified data from different experiments are presented as mean ± SD of three biological replicates. The *P-*values were calculated using a two-tailed Student’s *t-*test.

To assess the prevalence of pPAD8 in natural environments, we performed a homology search using the pPAD8 nucleotide sequence against the NCBI nonredundant nucleotide database. Sequences highly homologous to pPAD8 (83.47%–100% homology) were identified in 24 plasmids and one chromosome from multiple species, including *P. aeruginosa*, *Pseudomonas putida*, *Pseudomonas*sp., and *Pseudomonas juntendi* (Supplementary Table S4). Nearly all of these 24 plasmids share a similar backbone structure and carry a *dot*/*icm* T4BSS associated with conjugative transfer. Among these plasmids, pND6-2 and pS04 90 have been confirmed as conjugative plasmids through conjugative experiments [8, 43]. To investigate the potential conjugative transfer of pPAD8, a conjugation experiment was performed. In this experiment, PAD8, which carries an exogenous gentamicin resistance (Gm^R^) marker on pPAD8, was used as the donor strain, while PAO1, which carries a fosfomycin resistance marker generated by deleting the *glpT* gene from the chromosome, was used as the recipient strain (Fig. 1A). Clones grown under selection with gentamicin and fosfomycin were transconjugants (Supplementary Fig. S1B), confirming that pPAD8 can transfer from PAD8 to PAO1. Further experiments demonstrated that pPAD8 failed to transfer to *E. coli* (data not shown). These results indicate that pPAD8 is a conjugative plasmid with a narrow host range.

### Plasmid pPAD8 encodes an inactive type II TA system MtvTA

Two putative type II TA modules, *dinJ*/*yafQ* and *mtvTA*, were identified on the pPAD8 plasmid using TAfinder, a newly developed online tool in the TADB 2.0 database (http://bioinfo-mml.sjtu.edu.cn/TADB2/). To investigate the functions of *dinJ*/*yafQ* and *mtvTA* TA systems, we deleted each system from the pPAD8 plasmid. Interestingly, the deletion of the *dinJ*/*yafQ* system did not impact PAD8 bacterial cell growth, whereas the absence of the *mtvTA* system resulted in severe growth inhibition (Fig. 1B). In the present study, we first focused on investigating the biological function of the MtvTA system.

To determine whether the *mtvTA* system encodes a functional TA system, the toxicity of MtvT was evaluated by overexpressing *mtvT* in PAO1 and *E. coli* DH5α. Surprisingly, overexpression of *mtvT* did not result in growth inhibition or cell death (Supplementary Fig. S2A and B), suggesting that MtvT is nontoxic. Protein identifiers demonstrated that MtvT shares 32.41% amino acid sequence identity with the toxin MazF protein from the reported *mazE*/*mazF* TA in *E. coli* K-12 (*E_C_*MazF) (Supplementary Fig. S2C). A previous study revealed that Glu24 (E24) of *E_C_*MazF is an active residue responsible for its toxicity [44]. For MtvT, the corresponding position is Ala (A). To test whether the Ala residue is responsible for the non-toxicity of MtvT, a point mutant was introduced at position 24 by substituting Ala with Glu (MtvT^A24E^). Unexpectedly, the overexpression of MtvT^A24E^ in PAO1 still did not result in growth inhibition (Supplementary Fig. S2D), suggesting that the non-toxicity of MtvT may involve new mechanisms.

Type II TA system genes are typically organized into an operon, with the antitoxin gene usually located upstream of the toxin gene [45]. In *mtvTA* module, the coding regions of both genes were overlapped by four bases, with the *mtvA* gene containing the start codon of the *mtvT* gene (Fig. 1C). RT-PCR analysis confirmed that *mtvA* and *mtvT* are co-transcribed and organized as an operon (Fig. 1C). A GST pull‐down assay and a bacterial two‐hybrid (BATCH) assay together demonstrated that MtvA dose indeed interact with MtvT (Fig. 1D and E). In addition, the SDS–PAGE analysis confirmed that MtvT is co-eluted with His-tagged MtvA when purified with a Ni^2^-affinity column (Supplementary Fig. S2E).

Since most type II TA operons are autoregulated by the antitoxin alone or the TA complex, we further investigated the autoregulation of the *mtvTA* operon. Promoter activity assays were performed using the *mtvTA-lux* plasmid as the reporter. The results showed that the *mtvTA* promoter activity was significantly decreased upon the overexpression of MtvA and MtvTA in PAD8 (Fig. 1F), indicating that both MtvA and the MtvTA complex repress promoter activity. EMSA further demonstrated that MtvA efficiently binds and shifts its own promoter region, located from −593 to −293 relative to the TTG start codon of *mtvA* (P_1_-*mtvTA*), in a dose-dependent manner (Supplementary Fig. S3A). Additionally, the MtvTA complex was also shown to bind the promoter with higher sensitivity compared to MtvA alone (Fig. 1G). To identify the specific binding motif of MtvA, a series of truncated DNA fragments of the promoter were analyzed. The results demonstrated that a palindrome sequence (5′-TAACATTGTTA-3′) within a region from −493 to −302 in the promoter is the specific binding motif (Supplementary Fig. S3B). Furthermore, when the sequence TAACATTGTTA was mutated to **CTTG**ATTGTTA (mutated nucleosides underlined), no shifts were observed (Supplementary Fig. S3C), indicating that this motif is critical for MtvA and MtvTA binding. These results showed that both MtvA and the MtvTA complex can bind to the *mtvTA* promoter region and negatively autoregulate its own operon. Collectively, these results clearly reveal that the MtvTA system is an inactive type II TA system, in which the putative toxin MtvT exhibits no toxic properties.

### MtvA binds DNA and MtvT via a distinct domain

**Figure 2.**
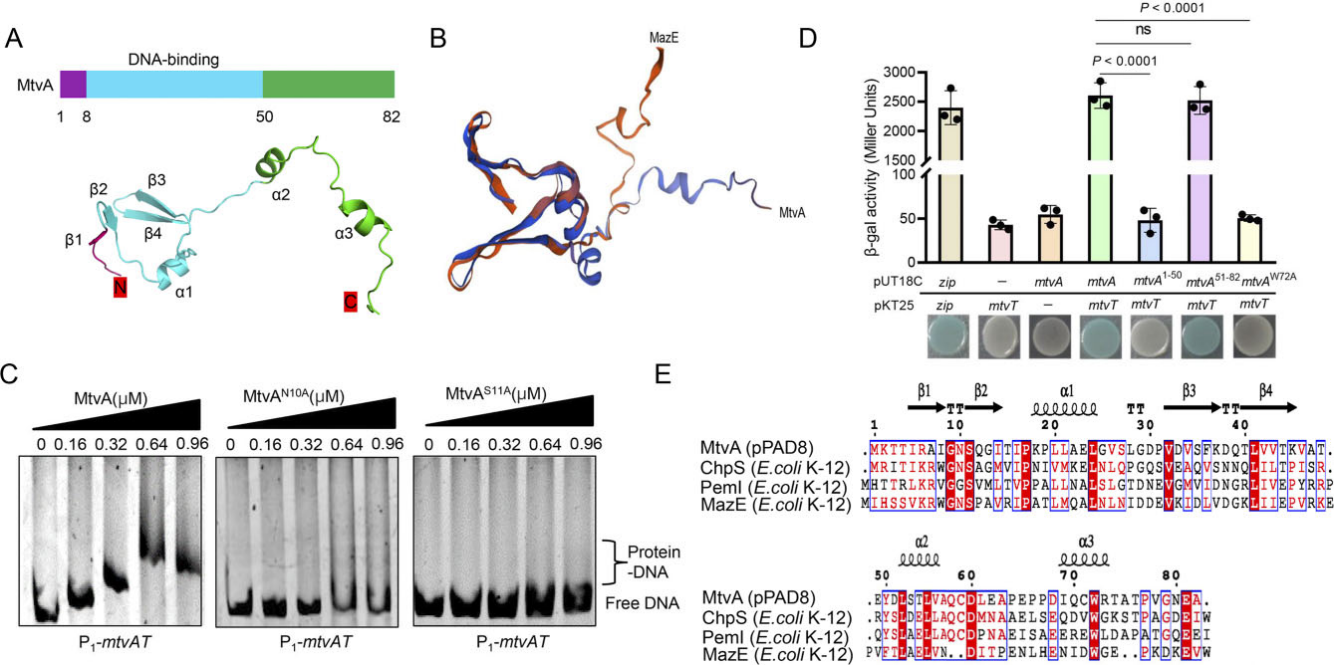
MtvA exhibits a bifunctional modular structure. (**A**) Schematic diagram of the domain architecture of protein MtvA on top. The 3D structure of MtvA is predicted by AlphaFold 2; DNA-binding domain is between residues 8 and 50. (**B**) Superposition of the MtvA structure and the antitoxin *Ec*MazE structure (PDB ID: 1UB4.1). (**C**) EMSA showing that MtvA^N10A^ and MtvA^S11A^ do not bind and shift P_1_-*mtvTA*. The binding of MtvA and P_1_-*mtvTA* was used as a positive control. Each reaction mixture contains 1.0 ng/μl of PCR products of P_1_-*mtvTA*. Protein concentrations are indicated above the lane. (**D**) A BACTH assay and β-galactosidase assay were performed to assess interactions between MtvA, MtvA mutants, and MtvT. The quantified data from different experiments are presented as mean ± SD of three biological replicates. The *P-*values were calculated by two-tailed Student’s *t-*test. (**E**) Multiple sequence alignment of the amino acid sequences of MtvA, ChpS (*E coli* K-12), PemI (*E coli* K-12), and MazE (*E coli* K-12) constructed using ClustalW. Conserved amino acids are highlighted in shaded boxes.

Structure predictions revealed that the N-terminal domain of MtvA functions as a DNA-binding domain and adopts a swapped-hairpin β-strand motif, which is characteristic of the AbrB/MazE superfamily (Fig. 2A and Supplementary Fig. S4A). Homology analysis revealed that MtvA shares 23.08% amino acid sequence identity with the antitoxin MazE from *E. coli* K-12 (*E_C_*MazE, PDB ID: 1UB4.1). Notably, the N-terminal structure of MtvA highly overlaps with that of the *E_C_*MazE (Fig. 2B). The N-terminal DNA-binding domain of *E_C_*MazE plays a key role in its function as a transcriptional regulator [46]. Therefore, to further investigate the key residues in the N-terminal of MtvA that are involved in DNA binding, molecular docking simulations were performed between MtvA and the palindromic sequence 5′-GGTAATAACATTGTTACTACC-3′ from the P_1_-*mtvTA* promoter. The results showed that four residues Asn10 (N10), Ser11 (S11), Thr48 (T48), and Tyr50 (Y50) may be involved in the interaction between MtvA and the P_1_-*mtvTA* promotor (Supplementary Fig. S4B). To confirm this, EMSA were performed using MtvA mutants (N10A, S11A, T48A, and Y50A). The results show that MtvA^N10A^ and MtvA^S11A^ did not bind and shift the P_1_-*mtvTA* promoter (Fig. 2C).

To determine whether the C-terminal region of MtvA is responsible for binding to MtvT, we constructed two truncated forms of MtvA, MtvA^1–50^ and MtvA^51–82^. Using a BATCH assay, we found that MtvA^51–82^ binds to MtvT as efficiently as full-length MtvA, whereas MtvA^1–50^ does not (Fig. 2D). To further identify the key residues in the C-terminal region of MtvA involved in MtvT binding, we compared the sequences of MtvA with those of other antitoxins from the MazE superfamily, including ChpS, PemI, and MazE from *E. coli* K-12. Sequence alignment revealed that six residues, Leu52 (L52), Leu55 (L55), Gln58 (Q58), Cys59 (C59), Asp60 (D60), and Trp72 (W72), are highly conserved in the C-terminal region of these four antitoxins (Fig. 2E). We then mutated these six conserved residues to alanine and performed a BATCH assay. The results showed that the point mutants MtvA^L52A^, MtvA^L55A^, MtvA^Q58A^, MtvA^C59A^, and MtvA^D60A^ interact with MtvT as efficiently as wild-type MtvA (Supplementary Fig. S4C). However, MtvA^W72A^ failed to interact with MtvT (Fig. 2D), indicating that W72 is a critical residue for the MtvA–MtvT interaction. Collectively, these results demonstrate that MtvA has a bifunctional module organization, with an N-terminal DNA-binding domain and a C-terminal MtvT-binding domain.

### MtvTA negatively regulates the conjugative transfer of pPAD8

**Figure 3.**
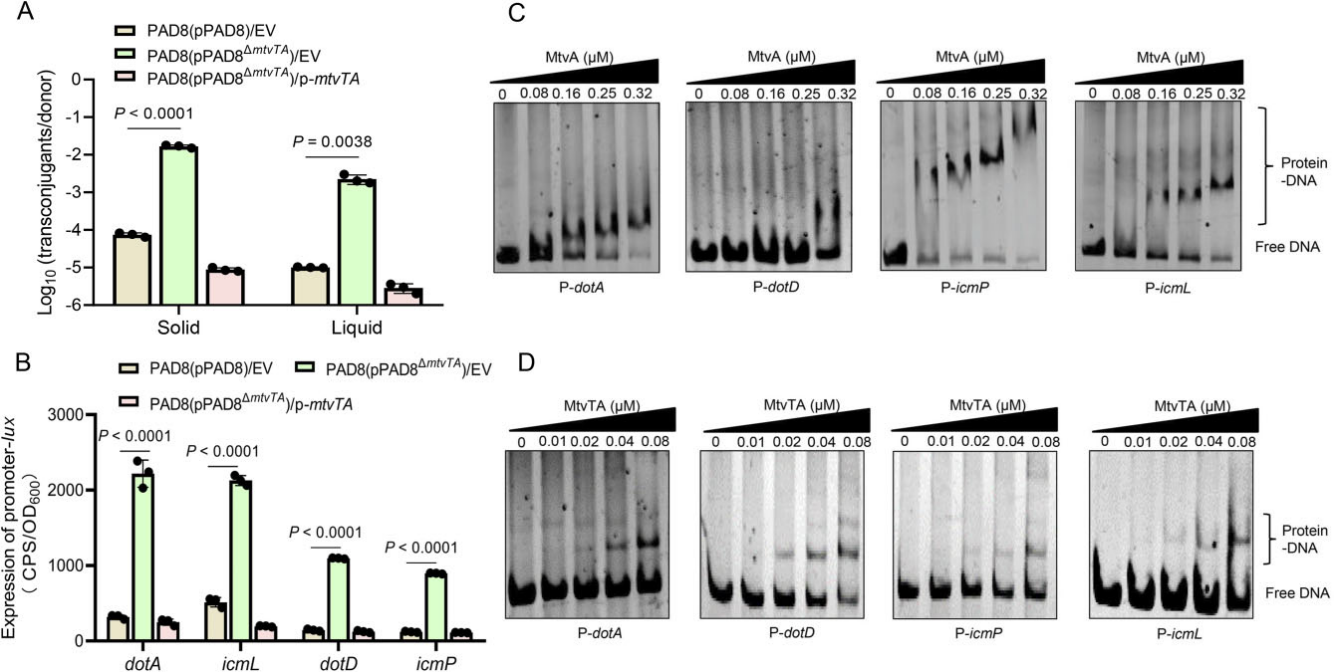
MtvTA controls plasmid transfer by negatively regulating the *dot*/*icm* system. (**A**) The conjugative transfer efficiency of the plasmid in wild-type PAD8(pPAD8), the mutant PAD8(pPAD8^Δ^*^mtvTA^*), and complemented strain was detected under different conditions. Conjugation assays conducted on solid plates are shown on the left, while those performed overnight in LB broth are shown on the right. EV represents empty vector pMMB67EH. (**B**) The promoter activity of *dotA*, *icmL*, *dotD*, and *icmP* was measured in the wild-type PAD8(pPAD8), the mutant PAD8(pPAD8^Δ^*^mtvTA^*), and its complemented strain. EV represents empty vector pMMB67EH. OD_600_, optical density at 600 nm. CPS, counts per second. (**C**) EMSA showing that MtvA binds and shifts the promoters P-*dotA*, P-*dotD*, P-*icmP*, and P-*icmL*. Each reaction mixture contains 1.0 ng/μl of PCR products of P-*dotA*, P-*icmP*, P-*icmL*, and P-*dotD*. (**D**) EMSA showing that the MtvTA complex binds and shifts the promoters P-*dotA*, P-*dotD*, P-*icmP*, and P-*icmL*. Each reaction mixture contains 1.0 ng/μl of PCR products of P-*dotA*, P-*icmP*, P-*icmL*, and P-*dotD*. The protein concentrations are indicated above the lane. For panels (A) and (B), the quantified data from different experiments are presented as mean ± SD of three biological replicates. The *P-*values were calculated by two-tailed Student’s *t-*test.

Sequence alignment revealed that the *dot*/*icm* system coexists with the MtvTA module in both pPAD8 and its homologous plasmid (Supplementary Fig. S5A). Plasmid pPAD8 encodes four *dot*/*icm* system gene clusters, *icmP*-*icmO*, *dotA*, *dotDCB*-*icmT*, and *icmLKEGCJB*. RT-PCR analysis further confirmed that these four *dot*/*icm* gene clusters are transcribed as independent operons (Supplementary Fig. S5B). As *dot*/*icm* systems are associated with HGT [47], we hypothesized that the MtvTA system may be involved in the conjugative transfer of plasmid pPAD8. To test this hypothesis, we measured the conjugative transfer efficiency of pPAD8 and pPAD8^Δ^*^mtvTA^* from PAD8 to PAO1 in solid and liquid media, respectively. Surprisingly, the transfer frequency of pPAD8^Δ^*^mtvTA^* showed a notable increase compared to pPAD8, increasing from 7.4 × 10^−5^ to 1.14 ×10^−2^ in solid medium (Fig. 3A). The complementation of *mtvTA* in PAD8(pPAD8^Δ^*^mtvTA^*) resulted in a more significantly decreased transfer frequency. The conjugative transfer efficiency was slightly lower in liquid medium than in solid medium, but the trend remained the same. These results indicate that the MtvTA system represses the conjugative transfer of plasmid pPAD8.

To further explore whether the MtvTA system represses the conjugative transfer of pPAD8 via the *dot*/*icm* system, the expression of *dot*/*icm* genes in PAD8(pPAD8) and PAD8(pPAD8^Δ^*^mtvTA^*) was quantified using qRT-PCR. As expected, *dot*/*icm* gene expression was significantly upregulated in the mutant PAD8(pPAD8^Δ^*^mtvTA^*) (Supplementary Fig. S5D). In addition, promoter activity of four operons, *dotA-lux*, *icmL-lux*, *dotD-lux*, and *icmP-lux*, was measured in PAD8(pPAD8), PAD8(pPAD8^Δ^*^mtvTA^*), and its complemented strain. As expected, the promoter activities of all four operons were drastically increased in the mutant PAD8(pPAD8^Δ^*^mtvTA^*) compared to both the wild-type PAD8(pPAD8) and the complemented strain (Fig. 3B). EMSAs revealed that both MtvA and the MtvTA complex efficiently bind to the promoter regions of *dotA*, *icmP*-*icmO*, *dotDCB*-*icmT*, and *icmLKEGCJB*, respectively (Fig. 3C and D). Collectively, these results demonstrate that MtvTA negatively modulates the conjugative transfer of pPAD8 by directly regulating the *dot*/*icm* system.

### The deletion of MtvTA attenuates the pathogenicity of its host strain

**Figure 4.**
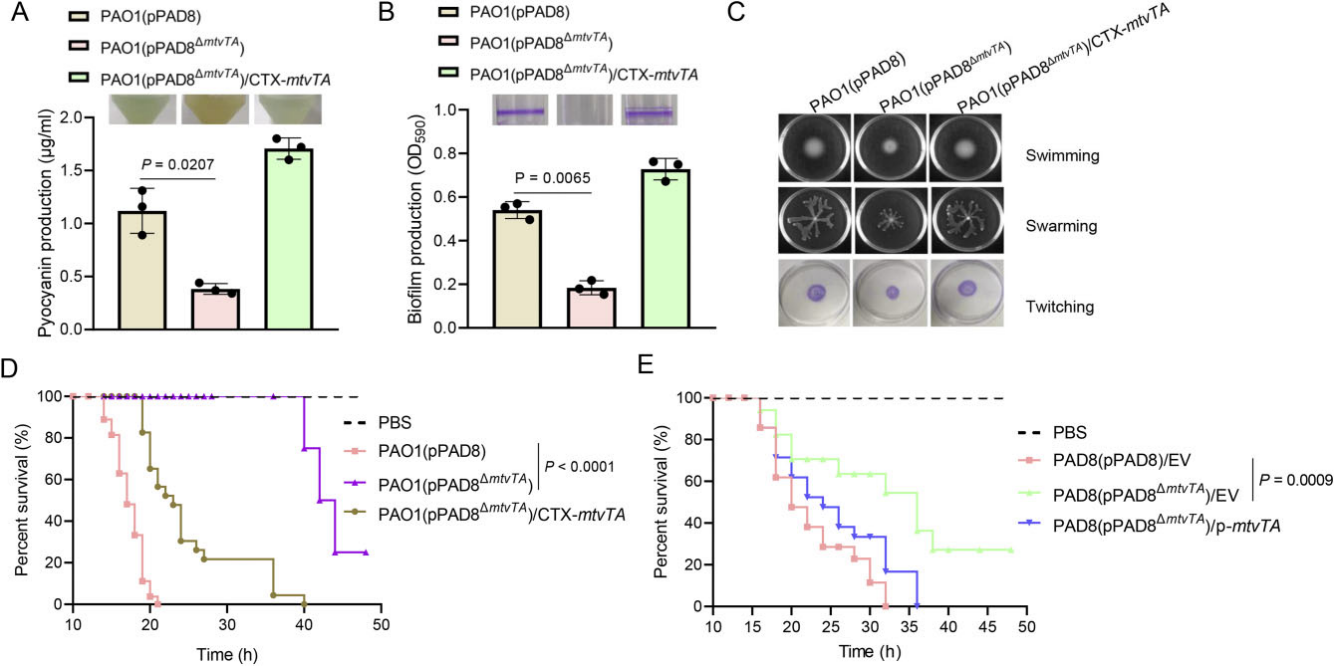
MtvTA alters *P*. *aeruginosa* PAO1 phenotypes and pathogenicity. (**A**) The pyocyanin production of the wild-type PAO1(pPAD8), the mutant PAO1(pPAD8^Δ^*^mtvTA^*), and its complemented strain was detected after culture in LB medium for 12 h. (**B**) The biofilm formation of the wild-type PAO1(pPAD8), the mutant PAO1(pPAD8^Δ^*^mtvTA^*), and its complemented strain was displayed with CV staining (top) and quantified with optical density measurement (bottom). (**C**) Motility assays: overnight cultures were spotted onto swimming, swarming, and twitching plates, followed by incubation at 30°C for 16 h (swimming) and 37°C for 16 h (swarming and twitching), respectively. (**D**) Virulence of the wild-type PAO1(pPAD8), the mutant PAO1(pPAD8^Δ^*^mtvTA^*), and its complemented strain was measured using a *G. mellonella* infection model. Each *G. mellonella* was injected with 20 μl of *P. aeruginosa* dilution (1 × 10^4^ CFU/ml) or PBS (negative control). (**E**) Virulence of the wild-type PAD8(pPAD8), the mutant PAD8(pPAD8^Δ^*^mtvTA^*), and its complemented strain was measured using *G. mellonella* infection model. EV represents empty vector pMMB67EH. For panels (A) and (B), the quantified data from different experiments are presented as mean ± SD of three biological replicates. The *P-*values were calculated by a two-tailed Student’s *t-*test. For panels (D) and (E), the quantified data from different experiments are presented as mean ± SD. The *P-*values were calculated by Mantel–Cox test. *n* = 20 biological replicates.

We observed that when the plasmid pPAD8^Δ^*^mtvTA^* was transferred to strain PAO1, cell growth was unaffected (Supplementary Fig. S6A), but the cultures turned more yellow compared to PAO1 carrying plasmid pPAD8 (Fig. 4A). However, the deletion of *mtvTA* did not induce any color changes in the original host strain PAD8 (data not shown). This color change was likely caused by variations in pyocyanin production, which typically imparts a bluish-green tint to the culture medium [48]. Consequently, pyocyanin production, biofilm formation, and motility were evaluated. Results demonstrated that PAO1(pPAD8^Δ^*^mtvTA^*) exhibited a significant decrease in pyocyanin production, biofilm formation, and motility compared to wild-type PAO1(pPAD8) (Fig. 4A–C). Complementation of *mtvTA* in PAO1(pPAD8^Δ^*^mtvTA^*) restored all phenotypes to the levels observed in PAO1(pPAD8).

Pyocyanin and biofilm are important virulence factors for *P. aeruginosa*. Additionally, type II antitoxins are known to be global regulators involved in multiple cellular processes through their DNA-binding domains [20, 24, 49, 50]. Therefore, we speculated that the MtvTA module may be involved in the pathogenicity of the host strain. In a *G. mellonella* infection model, we assessed the pathogenicity of the MtvTA deletion mutant in both the PAO1 and PAD8 host strains. As shown in Fig. 4D and E, PAO1(pPAD8^Δ^*^mtvTA^*) and PAD8(pPAD8^Δ^*^mtvTA^*) exhibited significantly reduced mortality rates compared to PAO1(pPAD8), PAD8(pPAD8), and their complemented strains, respectively. Collectively, these results suggest that MtvTA plays a key role in regulating *P. aeruginosa* virulence.

### Transcriptomic analysis of host strain carrying plasmid pPAD8 and pPAD8^Δ^*^mtvTA^*

To further explore the global impact of the MtvTA module, we performed transcriptome sequencing on strains carrying plasmid pPAD8 and pPAD8^Δ^*^mtvTA^*. DESeq2 was used to determine the significance of DEGs between PAD8(pPAD8) and PAD8(pPAD8^Δ^*^mtvTA^*), and between PAO1(pPAD8) and PAO1(pPAD8^Δ^*^mtvTA^*) according to a threshold of adjusted *P* < .05 and |log_2_fold change| > 1.

**Figure 5.**
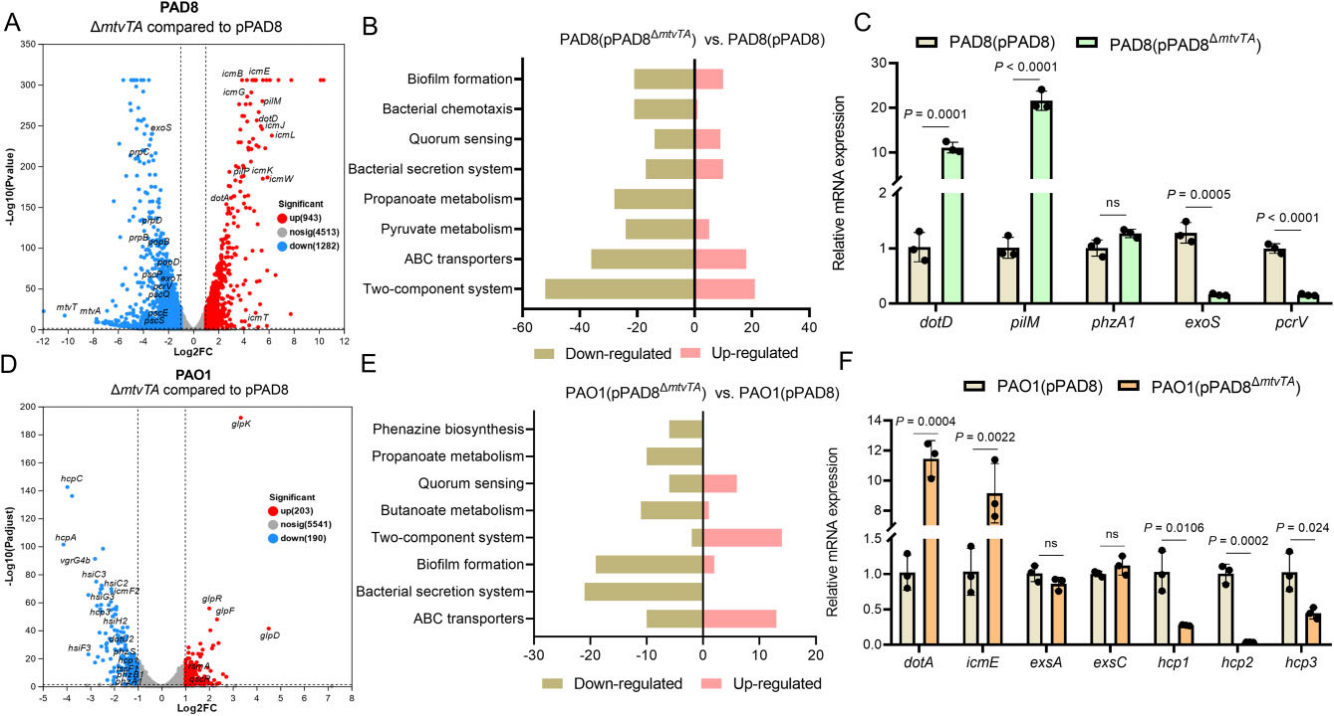
Transcriptomic analysis of the host strain carrying plasmid pPAD8 and pPAD8^Δ^*^mtvTA^*. (**A**) Volcano plot of the DEGs was analyzed between the wild-type PAD8(pPAD8) and the mutant PAD8(pPAD8^Δ^*^mtvTA^*) by RNA-seq. Every dot in the figure denotes a gene. (**B**) Pathway analysis by mapping DEGs to the KEGG pathway shows the upregulated and downregulated signaling pathways in PAD8(pPAD8^Δ*mtvTA*^) compared to PAD8(pPAD8). (**C**) The relative mRNA levels of *dotD*, *pilM*, *phzA1*, *exoS*, and *pcrV* were determined in the wild-type PAD8(pPAD8) and the mutant PAD8(pPAD8^Δ^*^mtvTA^*) by qRT-PCR. (**D**) Volcano plots of the DEGs were analyzed between the wild-type PAO1(pPAD8) and the mutant PAO1(pPAD8^Δ^*^mtvTA^*) by RNA-seq. (**E**) Pathway analysis by mapping DEGs to the KEGG pathway shows the upregulated and downregulated signaling pathways in PAO1(pPAD8^Δ*mtvT*A^) compared to PAO1(pPAD8). (**F**) The relative mRNA levels of *dotA*, *icmE*, *exsA*, *exsC*, *hcp1*, *hcp2*, and *hcp3* were determined in the wild-type PAO1(pPAD8) and the mutant PAO1(pPAD8^Δ^*^mtvTA^*) by qRT-PCR.

RNA-seq data revealed that the deletion of the *mtvTA* operon significantly altered gene expression in PAD8(pPAD8^Δ^*^mtvTA^*), with 943 genes upregulated and 1282 genes downregulated compared to PAD8(pPAD8) (Fig. 5A). The potential functions of these DEGs were analyzed using the KEGG pathway. The results indicate that these DEGs are involved in various processes, including the two-component system, pyruvate metabolism, bacterial secretion systems, and nitrogen metabolism (Fig. 5B). Notably, the repressed transcripts in PAD8(pPAD8^Δ^*^mtvTA^*) included genes associated with the T3SS system, such as *exos*, *exoT*, *pscN*, *popN*, *pcrG*, and *exsD* (Fig. 5A). In contrast, the increased transcripts included genes from the *dot*/*icm* system, such as *dotA*, *dotD*, *icmP*, and *icmLKEGCJB* (Fig. 5A), which is consistent with the findings described above, further supporting the conclusion that MtvTA negatively regulates the *dot*/*icm* system. To verify the reliability of the RNA-seq data, expression of randomly selected genes was analyzed by qRT-PCR. The expression of these selected genes was consistent with the RNA-seq results (Fig. 5C).

Similarly, the deletion of the *mtvTA* module significantly altered the expression of many genes, with 203 genes upregulated and 190 genes downregulated in the mutant PAO1(pPAD8^Δ^*^mtvTA^*) compared to PAO1(pPAD8) (Fig. 5D). These DEGs are involved in processes, such as the bacterial secretion system, biofilm formation, two-component system, QS, and phenazine biosynthesis (Fig. 5E). Notably, the expression levels of nearly all T6SS system genes were remarkably lower in PAO1(pPAD8^Δ^*^mtvTA^*), including genes from the H1-T6SS (*hcp1* and *tssF1*), H2-T6SS (*clpV2*, *hsiC2*, *icmF2*, and *fha2*), and H3-T6SS (*hsiF3*, *hcp3*, *clpV3*, and *siH3*) families (Fig. 5D). Additionally, genes associated with pyocyanin production, such as *phzA1* and *phzB1*, were significantly decreased in PAO1(pPAD8^Δ^*^mtvTA^*) (Fig. 5D). To verify the reliability of the RNA-seq data, expression of randomly selected genes was analyzed by qRT-PCR. The expression of these selected genes was consistent with the RNA-seq results (Fig. 5F). These results suggest that the MtvTA module is involved in various regulatory processes in the host strain. However, distinct gene expression profiles influenced by the MtvTA module were observed in PAD8 and PAO1, possibly due to the genetic background differences between the two host strains.

### MtvTA upregulates T3SS through the direct activation of *exsA* in PAD8

**Figure 6.**
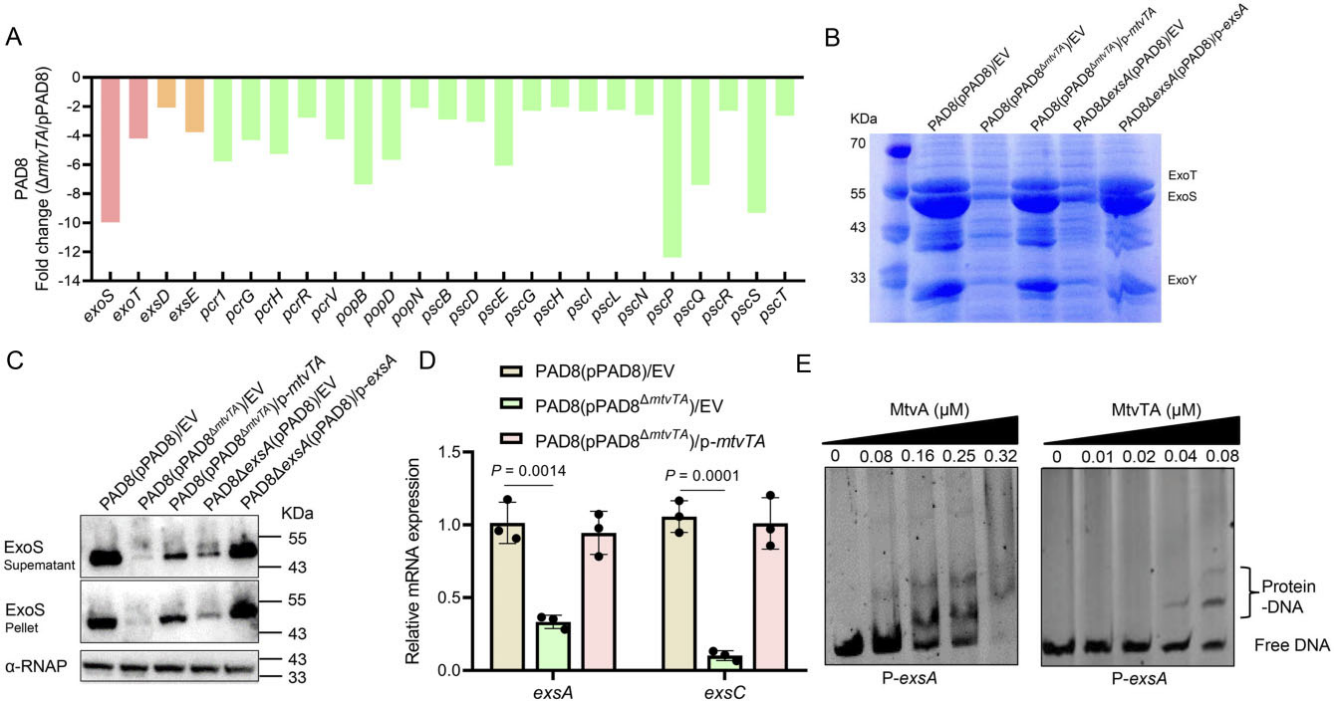
MtvTA upregulates T3SS through the direct activation of *exsA*. (**A**) Fold changes of the mRNA levels of the T3SS genes in the mutant PAD8(pPAD8^Δ^*^mtvTA^*) versus PAD8(pPAD8) from RNA-seq data. (**B**) Secreted T3SS effectors in T3SS-inducing medium analyzed by SDS–PAGE. Culture supernatants of various strains after 6 h of growth in T3SS inducing medium were concentrated and analyzed by SDS–PAGE, followed by staining with Coomassie blue. EV represents empty vector pMMB67EH. (**C**) The fractionated proteins were transferred to a nitrocellulose membrane and probed with a specific antibody to confirm the identity of ExoS. RNA polymerase α (α-RNAP) antibody was used as a loading control. EV represents the empty vector pMMB67EH. (**D**) The relative mRNA levels of *exsA* and *exsC* were determined in the wild-type PAD8(pPAD8), the mutant PAD8(pPAD8^Δ^*^mtvTA^*), and its complemented strain by qRT-PCR. The quantified data from different experiments are presented as mean ± SD of three biological replicates. The *P-*values were calculated by two-tailed Student’s *t-*test. EV represents the empty vector pMMB67EH. (**E**) EMSA showing that both MtvA and the MtvTA complex bind and shift the promoter region P-*exsA*. Each reaction mixture contains 1.0 ng/μl of PCR products of P-*exsA*. The protein concentrations are indicated above the lane.

Our RNA-seq data suggested that the deletion of *mtvTA* operon reduces T3SS expression in PAD8 (Fig. 6A). To confirm this finding, we assessed the production of the T3SS effectors, ExoT, ExoS, and ExoY in the wild-type strain PAD8(pPAD8), mutant PAD8(pPAD8^Δ^*^mtvTA^*), and complemented strain under T3SS-inducing conditions (5 mM Ethylene Glycol Tetraacetic Acid (EGTA) and 20 mM MgCl_2_). The PAD8Δ*exsA* mutant, which does not produce T3SS effectors, was used as a control. SDS–PAGE analysis revealed a significant decrease in the production of ExoT (53 kDa), ExoS (49 kDa), and ExoY (33 kDa) in the mutant PAD8(pPAD8^Δ^*^mtvTA^*) compared to the wild-type strain PAD8(pPAD8) and its complemented strain (Fig. 6B). Western blotting analysis confirmed the reduced ExoS expression in PAD8(pPAD8^Δ^*^mtvTA^*) (Fig. 6C). These results indicate that the deletion of MtvTA is responsible for the decreased secretion of the T3SS effector.

In *P. aeruginosa*, the T3SS master regulator ExsA autoregulates its own *exsCEBA* operon by activating *exsC* [51]. The qRT-PCR assay indicated that the mRNA levels of *exsA* and *exsC* were significantly lower in PAD8(pPAD8^Δ^*^mtvTA^*) than in PAD8(pPAD8) and the complemented strain PAO1(pPAD8^Δ^*^mtvTA^*)/p-*mtvTA* (Fig. 6D). Using an EMSA indicated that both MtvA and the MtvTA complex efficiently bind to the promoter region of *exsA* (P-*exsA*), but not to that of *exsC* (P-*exsC*) (Fig. 6E and Supplementary Fig. S6B). These results demonstrate that MtvA and the MtvTA complex directly activate the transcription of *exsA* by binding to its promoter region. Altogether, these results suggest that MtvTA upregulates T3SS through the direct activation of *exsA* in PAD8.

### MtvTA upregulates T6SS gene expression by directly regulating *rsmY* and *rsmZ* in PAO1

**Figure 7.**
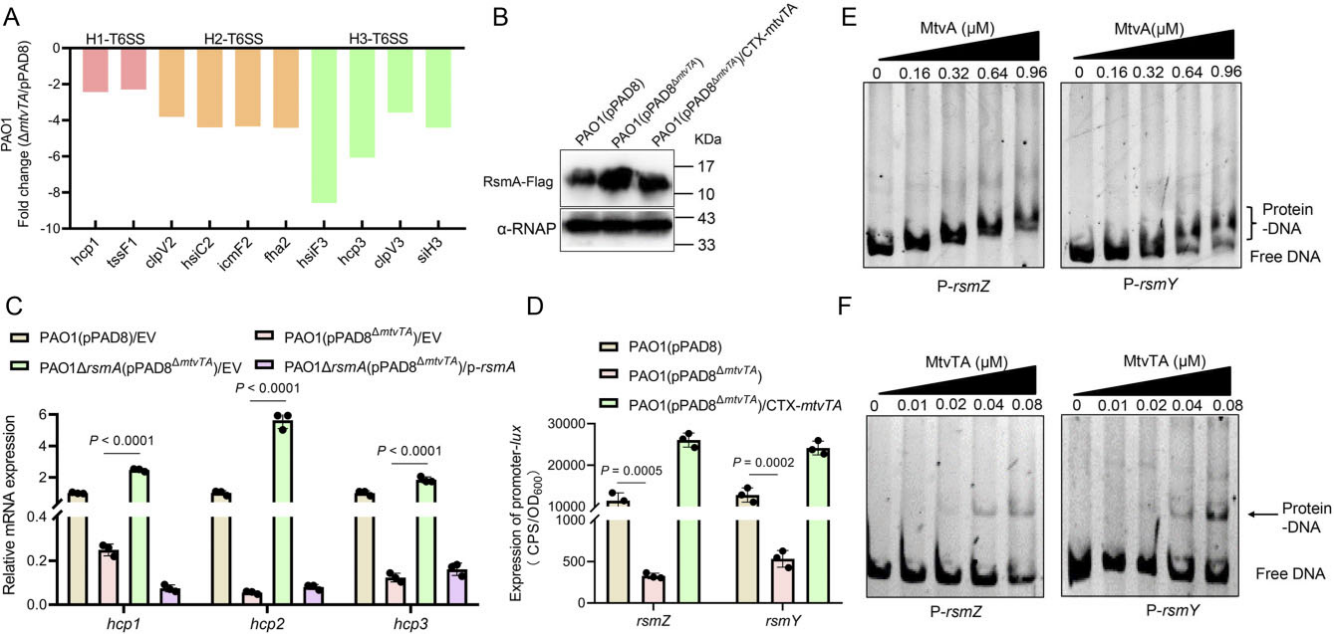
MtvTA upregulates T6SS through the direct regulation of *rsmY* and *rsmZ*. (**A**) Fold changes of the mRNA levels of the H1-T6SS, H2-T6SS, H3-T6SS genes in the mutant PAO1(pPAD8^Δ^*^mtvTA^*) versus PAO1(pPAD8) from RNA-seq data. (**B**) The protein level of RsmA-Flag was detected in the wild-type PAO1(pPAD8), the mutant PAO1(pPAD8^Δ^*^mtvTA^*), and its complemented strain by western blot. The tagged proteins were detected using a Flag antibody. α-RNAP antibody was used as a loading control. (**C**) The relative mRNA levels of *hcp1*, *hcp2*, and *hcp3* were determined in the wild-type PAO1(pPAD8), the mutant PAO1(pPAD8^Δ^*^mtvTA^*), the double mutant PAO1Δ*rsmA*(pPAD8^Δ^*^mtvTA^*), and *rsmA*-complemented strain by qRT-PCR. EV represents empty vector pAK1900. (**D**) The promoter activity of *rsmY* and *rsmZ* was measured in the wild-type PAO1(pPAD8), the mutant PAO1(pPAD8^Δ^*^mtvTA^*), and its complemented strain. EV represents empty vector pAK-1900. OD_600_, optical density at 600 nm. CPS, counts per second. (**E**) EMSA showed that MtvA binds and shifts the promoters P-*rsmY* and P-*rsmZ*. Each reaction mixture contains 1.0 ng/ml of PCR products of P-*rsmY* and P-*rsmZ*. The protein concentrations are indicated above the lane. (**F**) EMSA showed that the MtvTA complex binds and shifts the promoters P-*rsmY* and P-*rsmZ*. Each reaction mixture contains 1.0 ng/ml of PCR products of P-*rsmY* and P-*rsmZ*. The protein concentrations are indicated above the lane. For panels (C) and (D), the quantified data from different experiments are presented as mean ± SD of three biological replicates. The *P-*values were calculated by two-tailed Student’s *t-*test.

RNA-seq analysis also showed that the expression levels of almost all T6SS genes were significantly reduced in the PAO1(pPAD8^Δ^*^mtvTA^*) mutant compared to the wild-type PAO1(pPAD8) (Supplementary Table S5). The H2-T6SS (*clpV2*, *hsiC2*, *icmF2*, and *fha2*) and H3-T6SS (*hsiF3*, *hcp3*, *clpV3*, and *siH3*) genes were downregulated to a greater extent than the H1-T6SS (*hcp1* and *tssF1*) genes (Fig. 7A). Interestingly, the expression of *rmsA*, an important post-transcriptional regulator gene that negatively regulates the expression of all three T6SS clusters, was upregulated 2.7-fold in the PAO1(pPAD8^Δ^*^mtvTA^*) mutant compared to the wild-type PAO1(pPAD8) strain (Supplementary Fig. S6C). This finding was also confirmed by qRT-PCR (Supplementary Fig. S6C). Consistent with the transcription level of *rsmA*, western blot analysis further demonstrated a marked increase in RsmA protein levels in the PAO1(pPAD8^Δ^*^mtvTA^*) mutant compared to the wild-type PAO1(pPAD8) and the complemented strain (Fig. 7B). These results suggest that the MtvTA modulates the expression level of *rsmA*. To further explore whether MtvTA controls T6SS expression via RsmA, we constructed a double mutant PAO1Δ*rsmA*(pPAD8^Δ^*^mtvTA^*). RT-PCR analysis showed that the transcription levels of *hcp1*, *hcp2*, and *hcp3* were significantly higher in the double mutant than in PAO1(pPAD8^Δ^*^mtvTA^*). Moreover, these levels were significantly reduced upon the overexpression of *rsmA* in the double mutant (Fig. 7C). These results demonstrate that MtvTA upregulates T6SS expression by regulating RsmA.

Previous studies have shown that all three T6SSs in *P. aeruginosa* are regulated by the RetS/LadS–GacS/GacA–RsmY/RsmZ–RsmA pathway [31]. To determine whether MtvTA regulates T6SS expression through this pathway, we measured the mRNA levels of five upstream genes (*retS*, *ladS*, *gacA*, *rsmY*, and *rsmZ*) in PAO1(pPAD8), PAO1(pPAD8^Δ^*^mtvTA^*), and its complemented strain. qRT-PCR analysis showed that the mRNA levels of *rsmY* and *rsmZ* were significantly reduced in PAO1(pPAD8^Δ^*^mtvTA^*) compared to PAO1(pPAD8) and the complemented strain, whereas the expression of other genes did not differ between two strains (Supplementary Fig. S6D). In addition, the promoter activity of *rsmY* and *rsmZ* was significantly decreased in PAO1(pPAD8^Δ^*^mtvTA^*) compared to PAO1(pPAD8) and the complemented strain (Fig. 7D). EMSA confirmed that both MtvA and the MtvTA complex binds to the promoter regions *rsmY* (P-*rsmY*) and *rsmZ* (P-*rsmZ*) (Fig. 7E and F). These results indicate that MtvA and the MtvTA complex directly upregulate the expression of *rsmY* and *rsmZ*, both of which act as post-transcriptional repressors of *rsmA* [33]. Collectively, these results support a model in which MtvTA upregulates T6SS by downregulating the expression of the inhibitory factors *rsmY* and *rsmZ*.

### MtvTA indirectly regulates pyocyanin production via the *las* system in PAO1

**Figure 8.**
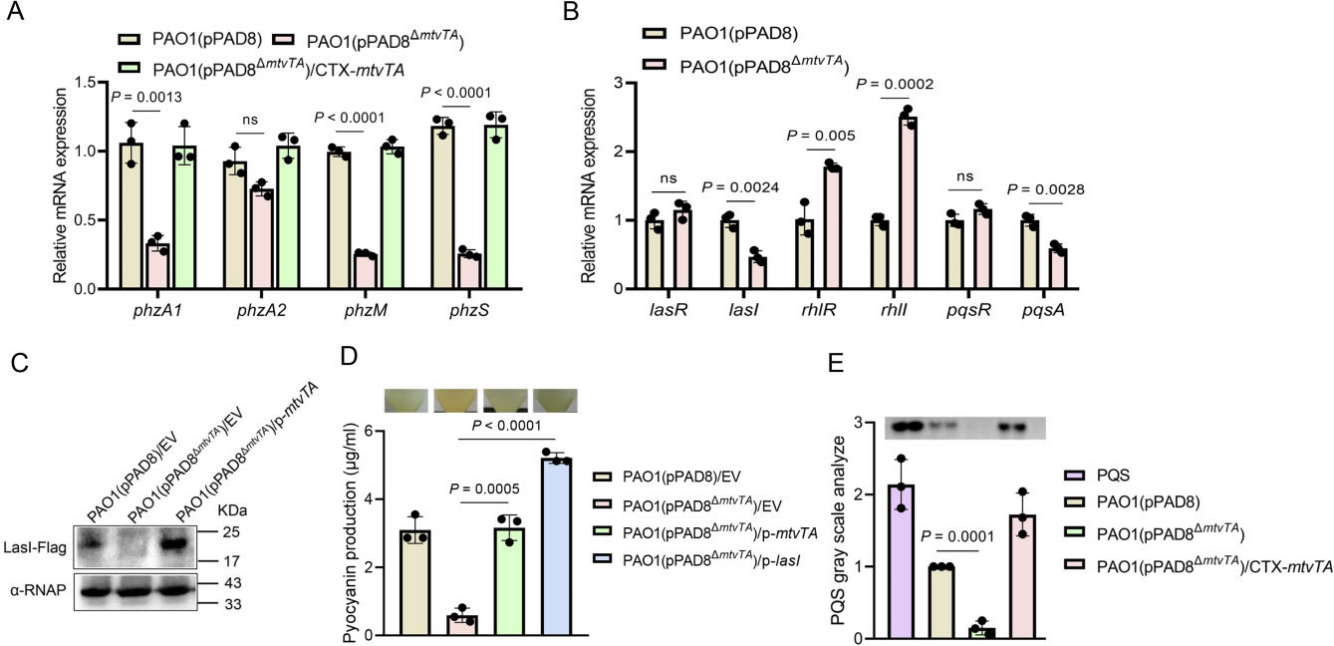
MtvTA controls pyocyanin production by indirectly regulating the QS system. (**A**) The relative mRNA levels of *phzA1*, *phzA2*, *phzM*, and *phzS* were determined in the wild-type PAO1(pPAD8), the mutant PAO1(pPAD8^Δ^*^mtvTA^*), and its complemented strain by qRT-PCR. (**B**) The relative mRNA levels of *lasR*, *lasI*, *rhlR*, *rhlI*, *pqsR*, and *pqsA* were determined in the wild-type PAO1(pPAD8) and the mutant PAO1(pPAD8^Δ^*^mtvTA^*) by qRT-PCR. (**C**) The protein level of LasI-Flag was detected in the wild-type PAO1(pPAD8), the mutant PAO1(pPAD8^Δ^*^mtvTA^*), and its complemented strain by western blot. EV represents the empty vector pAK1900. (**D**) The pyocyanin production of the wild-type PAO1(pPAD8), the mutant PAO1(pPAD8^Δ^*^mtvTA^*), the complemented strain, and *lasI*-overexpressed strain was detected after culture in LB medium for 12 h. EV represents the empty vector pAK1900. (**E**) TLC analysis of PQS produced by the wild-type PAO1(pPAD8), the mutant PAO1(pPAD8^Δ^*^mtvTA^*), and the complemented strain. PQS is a standard sample control. For panels (A), (B), (D), and (E), the quantified data from different experiments are presented as mean ± SD of three biological replicates. The *P-*values were calculated by two-tailed Student’s *t-*test.

As pyocyanin production was significantly lower when PAO1 harboring the plasmid pPAD8^Δ^*^mtvTA^* (Fig. 4A), we conducted qRT-PCR analysis of four genes associated with pyocyanin production and modification, *phzA1*, *phzA2*, *phzM*, and *phzS*. The results revealed that the transcription levels of *phzA1*, *phzM*, and *phzS* were significantly reduced in PAO1(pPAD8^Δ^*^mtvTA^*) compared to PAO1(pPAD8) (Fig. 8A). Since pyocyanin production is regulated by the QS system [52], we hypothesized that MtvTA might affect pyocyanin production by regulating the QS system. To test this hypothesis, the transcription levels of QS-related genes, *lasI*, *lasR*, *rhlI*, *rhlR*, *pqsR*, and *pqsA*, were analyzed using qRT-PCR. The result showed that the transcription levels of *lasI* and *pqsA* were significantly reduced, whereas those of *rhlI* and *rhlR* were increased in PAO1(pPAD8^Δ^*^mtvTA^*) (Fig. 8B). Western blotting analysis confirmed that the deletion of *mtvTA* dramatically decreased the expression of *lasI* (Fig. 8C). However, EMSA analysis revealed that MtvA and the MtvTA complex do not bind to or shift the promoter of *lasI* (P-*lasI*) (Supplementary Fig. S7A). In addition, overexpression of *lasI* in PAO1(pPAD8^Δ^*^mtvTA^*) restored pyocyanin production (Fig. 8D). These results indicate that MtvTA indirectly upregulates the *las* system.

Previous studies have shown that the *las* system is the top regulator within the QS hierarchy, positively regulating both the *rhl*and *pqs* systems [53–55]. However, our results suggest that MtvTA positively regulates the *las* system while negatively regulates the *rhl* system. Moreover, we found that the deletion of the *rhl* system did not affect pyocyanin production in PAO1(pPAD8^Δ^*^mtvTA^*) (data not shown). Since pyocyanin biosynthesis is directly regulated by the *pqs* system, we suspected that MtvTA might affect the production of the *pqs* system autoinducer 2-heptyl-3-hydroxy-4-quinolone (PQS) through the *las* system [53]. TLC analysis showed that PQS production was significantly reduced in PAO1(pPAD8^Δ^*^mtvTA^*) compared to PAO1(pPAD8) and the complemented strain (Fig. 8E). These results suggest that the MtvTA system affects pyocyanin production by interfering with the *pqs* system. Collectively, these results suggest that the MtvTA system influences pyocyanin production through the *las* and *pqs* systems.

## Discussion

In this study, we identified a MtvTA module, encoded on the plasmid pPAD8 from the clinical *P. aeruginosa* strain PAD8. MtvT shares 32.41% amino acid identity with *E_C_*MazF, a sequence-specific endoribonuclease that preferentially cleaves single-stranded mRNAs at either the 3′ or 5′ side of the first A in ACA sequences in a ribosome-independent manner [56]. However, under our experimental conditions, we did not detect MtvT toxicity to bacterial cells upon its overexpression in *P. aeruginosa* or *E. coli*. These results suggest that the MtvTA system might function as an active TA system. It is possible that the *mtvT* gene has undergone adaptive evolution, or that its toxin might require modification by other proteins. A previous study discovered that the *Burkholderia*-related microbe *Candidatus* Glomeribacter gigasporarum (*Ca*gg) possesses the ChpS/ChpB TA system, which is involved in the bacterial stress responses. However, ChpB also showed no effect on *E. coli* growth [57]. Therefore, MtvTA appears to be an inactive type II TA system, and its specific mechanism requires further study.

Type II TA systems are involved in diverse biological functions, including virulence and biofilm formation, antibiotic resistance, persistence formation, prophage production, plasmid maintenance, and plasmid replication [18, 45]. Here, we observed that MtvTA negatively modulates the conjugative transfer of pPAD8 by directly regulating *dotA*, *icmP*-*icmO*, *dotDCB*-*icmT*, and *icmLKEGCJB*. The deletion of MtvTA module resulted in high pPAD8^Δ^*^mtvTA^* transfer efficiency and reduced growth of the host strain PAD8. Usually, conjugation is energetically expensive, requiring substantial ATP for mating channel formation and plasmid DNA translocation [58]. Therefore, a large conjugative plasmid with high transfer rates generally imposes a significant cost burden on its host [59]. To minimize this cost, plasmids tightly regulate the conjugative system by repressing the expression of conjugation-related genes [3, 60, 61]. We hypothesized that the increased transfer efficiency of pPAD8^Δ^*^mtvTA^* might impose a burden on the PAD8 strain, leading to the reduced growth. However, pPAD8^Δ^*^mtvTA^* did not cause growth defects in PAO1, possibly due to its different genetic background compared to PAD8.

Structural analysis of MtvA revealed that it comprises an N-terminal DNA binding domain and a C-terminal MtvT binding domain. Indeed, in our study, we found that MtvTA functions as a multifaceted regulator, influencing several key biological processes. However, sequence analysis of all the promoter regions regulated by MtvA and the MtvTA complex reveals no significant sequence similarities, suggesting that the MtvTA module is a broad regulator. More significantly, our data revealed that MtvTA plays a pivotal role in modulating the pathogenicity of host strains. It positively regulates T3SS by directly binding to the *exsA* promoter region in the host strain PAD8, while it also positively regulates T6SS in the host strain PAO1 by directly binding to the promoter regions of *rsmY* and *rsmZ*. In *P. aeruginosa*, RsmA acts as a global translational regulatory protein, and its activity is inhibited by the small RNAs RsmY and RsmZ. RsmA activates T3SS while repressing T6SS [62]. Furthermore, a previous study showed that *rsmA* downregulates *lasI* and *rhlI* [63]. In the current study, we found that the transcription levels of *rsmY*, *rsmZ*, and *lasI* were decreased, whereas RsmA expression was increased, in the mutant PAO1(pPAD8^Δ^*^mtvTA^*) compared to PAO1(pPAD8) (Supplementary Fig. S6C). However, the deletion of *rsmA* did not restore the pyocyanin production in PAO1(pPAD8^Δ^*^mtvTA^*) (data not shown), suggesting that MtvTA regulates RsmA through RsmY and RsmZ, whereas the regulation of *lasI* is independent of RsmA in the host strain PAO1.

The MtvTA system altered different bacterial phenotypes by mediating distinct genes in two different host strains, PAD8 and PAO1, suggesting that the manipulation of bacterial gene regulation by the TA system is influenced by both the plasmid and the genetic background of the host strain. For example, the PCAR1 plasmid was introduced into three different *Pseudomonas* host strains (*P. putida* KT2440, *P. aeruginosa* PAO1, and *P. fluorescens* Pf0-1), resulting in different transcriptional effects under different host backgrounds [64, 65]. Similarly, in *Rhizobium favelukesii* LPU83, the genes *rcgA* and *rcgR* on the conjugative transfer plasmid pRfaLPU83a can activate and inhibit plasmid conjugative transfer, respectively. However, both genes had no effect on plasmid conjugative transfer in *Sinorhizobium meliloti* strain LPU88 [66]. These findings suggest that the genetic background of the host strain plays a crucial role in plasmid persistence and shapes the interactions between the plasmid and its host [67]. Thus, differential effects of the same plasmid in different bacterial strains may reflect the adaptive responses that allow bacterial cells to survive stresses or colonize new niches [65]. This hypothesis is consistent with our observation of differential gene regulation by MtvTA in different host strains.

Our data confirmed that MtvA binds strongly to MtvT (Supplementary Fig. S2E), and the MtvTA complex exhibits higher affinity for DNA than MtvA alone (Figs 1G, 3D, 6E, and 7F). Therefore, it is highly probable that MtvA and MtvT form a complex during the gene regulation process. This hypothesis provides an explanation of the similar high transfer efficiency of pPAD8 observed in PAD8(pPAD8^Δ^*^mtvT^*), PAD8(pPAD8^Δ^*^mtvA^*), and PAD8(pPAD8^Δ^*^mtvTA^*) (Fig. 3A and Supplementary Fig. S5C). Additionally, similar patterns of biofilm formation and pyocyanin production were observed in PAO1(pPAD8^Δ^*^mtvA^*), PAO1(pPAD8^Δ^*^mtvT^*), and PAO1(pPAD8^Δ^*^mtvTA^*) (Fig. 4A and B, and Supplementary Fig. S7B and C). These results suggest that the MtvTA complex plays key roles in plasmid conjugative transfer and bacterial virulence, rather than the individual MtvA or MtvT. Similar models are seen in the *E. coli* MazE–MazF, RelE–RelB, and DinJ–YafQ TA pairs [68–70].

In summary, we identified an inactive plasmid-encoded type II TA system, MtvTA, which influences plasmid conjugative transfer as well as a wide range of physiological activities in the host strain. The MtvTA complex acts as a negative regulator of conjugative transfer while positively influencing T3SS, T6SS, biofilm formation, pyocyanin biosynthesis, and motility. These findings shed light on the functional roles of large conjugative plasmids and their impact on host physiology, providing new insights into plasmid–host interactions.

## Supplementary Material

gkaf075_Supplemental_Files

## Data Availability

These genome sequences of the clinical *P. aeruginosa* isolate PAD8 and plasmid pPAD8 have been submitted to GenBank under accession numbers CP061073.2 and CP061074.2, respectively. The RNA-seq data for PAD8(pPAD8)/PAD8(pPAD8^Δ^*^mtvTA^*) and PAO1(pPAD8)/PAO1(pPAD8^Δ^*^mtvTA^*) are available in the NCBI BioProject database under accession numbers PRJNA1127266 and PRJNA1127263, respectively. All others supporting the findings of this study are available within the article and its supplementary materials.
