# Supplementary material for: A plasmid-encoded inactive toxin–antitoxin system MtvT/MtvA regulates plasmid conjugative transfer and bacterial virulence in Pseudomonas aeruginosa
Source: Nucleic Acids Res. 2025 Feb 14;53(4):gkaf075. doi: 10.1093/nar/gkaf075 (PMC11826091; doi:10.1093/nar/gkaf075)
Supplement: gkaf075_Supplemental_Files [file gkaf075_supplemental_files.zip › Supplementary Table.pdf]

Supplementary Table S1. Strains and plasmids used in this study.

Strain or plasmid	Relevant characteristics	Source
E.coli		
DH5a	*F⁻,φ80,lacZΔM15,Δ(lacZYA-argF)U169,endA1,recA1 hsdR17(rk–, mk+),supE44, thi-1,gyrA96 ,relA1,ponA*	Invitrogen
Transetta (DE3)	*F-,ompT,hsdS*$_B$*(r*$_B^-$*m*$_B^-$*)gal,dcm,lacY1(DE3) pRARE(argU,argW,ilex,glyT,leuW,proL)(Cam*^{r}*)*	Transgen
BTH101	*F*$^-$*, cya-99, araD139, galE15, galK16, rpsL1 (Str*^{r}*), hsdR2, mcrA1, mcrB1*	This lab
P. aeruginosa		
PAO1	Wild type model strain	This lab
PAD8(pPAD8)	Wild type clinical strain, containing conjugative plasmid pPAD8	This study
PAO1(pPAD8)	Wild type transconjugant, containing conjugative plasmid pPAD8	This study
Δ*mtvA*	*mtvA* deletion mutant of plasmid pPAD8; Gm^r	This study
Δ*mtvT*	*mtvT* deletion mutant of plasmid pPAD8; Gm^r	This study
Δ*mtvTA*	*mtvTA* deletion mutant of plasmid pPAD8; Gm^r	This study
Δ*dinJ/yafQ*	*dinJ*/*yafQ* deletion mutant of plasmid pPAD8; Gm^r	This study
PAO1Δ*glpT*	*glpT* deletion mutant of PAO1	This study
PAO1Δ*rsmA*(pPAD8$^{\Delta mtvTA}$)	*rsmA* deletion mutant of PAO1 (pPAD8$^{\Delta mtvTA}$); Gm^r	This study
**Plasmid**		
pPAD8	140 kb native plasmid in clinical *P. aeruginosa* PAD8	This study
pAK1900	*E. coli–P. aeruginosa* shuttle cloning vector carrying P*lac* upstream of MCS; Cb^r	This lab
mini-CTX-*lacZ*	Integration plasmid; Tc^r	This lab
pEX18Ap	*oriT*$^+$ *sacB*$^+$ gene replacement vector with multiple-cloning site from pUC18; Ap^r	This lab
pMS402	Expression reporter plasmid carrying the promoterless *luxCDABE* gene; Kn^r, Tmp^r	This lab

pET28a	Expression vector carrying T7 promoter; Kn^r	Novagen
pMMB67EH	*E. coli–P. aeruginosa* shuttle cloning vector; Ap^r, Cb^r	Novagen
pGEX-6p-1	Expression vector with N-terminal GST tag; Ap^r	Novagen
pUT18C	Bacterial two-hybrid assay plasmid, *lac* promoter and the T18 fragment for C-terminal heterologous protein fusion; Cb^r	This lab
pUT18C-*zip*	Leucine zipper of GCN1 cloned into pUT18C for BTH positive control; Cb^r	This lab
pKT25	Bacterial two-hybrid assay plasmid, *lac* promoter and the T25 fragment for C-terminal heterologous protein fusion; Km^r	This lab
pKT25-*zip*	Leucine zipper of GCN1 cloned into pKT25 for BTH positive control; Km^r	This lab
pBBR1MCS-5	Broad host shuttle vector; Gm^r	This lab
pEX18Ap-*mtvA*	*mtvA* deletion plasmid; Cb^r, Gm^r	This study
pEX18Ap-*mtvT*	*mtvT* deletion plasmid; Cb^r, Gm^r	This study
pEX18Ap-*mtvTA*	*mtvA-mtvT* double deletion plasmid; Cb^r, Gm^r	This study
pEX18Ap-*dinJ/yafQ*	*dinJ-yafQ* double deletion plasmid; Cb^r, Gm^r	This study
pEX18Ap-*glpT*	*glpT* deletion plasmid; Cb^r	This study
pEX18Ap-*rsmA*	*rsmA* deletion plasmid; Cb^r	This lab
pBBR1-*mtvA*	pBBR1MCS-5 derived plasmid expressing *mtvA*; Gm^r	This study
pBBR1-*mtvT*	pBBR1MCS-5 derived plasmid expressing *mtvT*; Gm^r	This study
pBBR1-*mtvTA*	pBBR1MCS-5 derived plasmid expressing *mtvTA*; Gm^r	This study
pAK-1900-*mtvTA*	pAK-1900 derived plasmid expressing *mtvTA*; Cb^r	This study
pAK-1900-*rsmA*	pAK-1900 derived plasmid expressing *rsmA*; Cb^r	This study
pAK-1900-*lasI*	pAK-1900 derived plasmid expressing *lasI*; Cb^r	This lab
mini-CTX-*mtvTA*	mini-CTX-*lacZ* containing *mtvTA* and its own promoter; Tc^r	This study
mini-CTX-*lasI*-Flag	mini-CTX-*lacZ* containing *lasI* promoter and the entire *lasI* gene fused with FLAG tag at C-terminal; Tc^r	This lab
pMMB67EH-*rsmA*-Flag	pMMB67EH containing the entire *rsmA* gene fused with FLAG tag at C-terminal; Cb^r	This study

pMMB67EH-*mtvTA*-Flag	pMMB67EH containing the entire *mtvTA* operon fused with FLAG tag at C-terminal; Cb^r	This study
pET28a-*mtvA*	pET28a derived plasmid for MtvA-His expression; Kn^r	This study
pET28a-$mtvA^{N10A}$	pET28a derived plasmid for $MtvA^{N10A}$-His expression; Kn^r	This study
pET28a-$mtvA^{S11A}$	pET28a derived plasmid for $MtvA^{S11A}$-His expression; Kn^r	This study
pET28a-$mtvA^{T48A}$	pET28a derived plasmid for $MtvA^{T48A}$-His expression; Kn^r	This study
pET28a-$mtvA^{Y50A}$	pET28a derived plasmid for $MtvA^{Y50A}$-His expression; Kn^r	This study
pET28a-$mtvA^{1\text{-}50}$	pET28a derived plasmid for $MtvA^{1\text{-}50}$-His expression; Kn^r	This study
pET28a-*mtvT*	pET28a derived plasmid for MtvAT-His expression; Kn^r	This study
pGEX6p-1-*mtvA*	pGEX6p-1 derived plasmid for GST- MtvA expression; Cb^r	This study
mtvTA-lux	pMS402 containing *mtvTA* promoter region; Kn^r, Tmp^r	This study
dotA-lux	pMS402 containing *dotA* promoter region; Kn^r, Tmp^r	This study
dotD-lux	pMS402 containing *dotD* promoter region; Kn^r, Tmp^r	This study
icmP-lux	pMS402 containing *icmP* promoter region; Kn^r, Tmp^r	This study
icmL-lux	pMS402 containing *icmL* promoter region; Kn^r, Tmp^r	This study
exsA-lux	pMS402 containing *exsA* promoter region; Kn^r, Tmp^r	This lab
exsC-lux	pMS402 containing *exsC* promoter region; Kn^r, Tmp^r	This lab
lasI-lux	pMS402 containing *lasI* promoter region; Kn^r, Tmp^r	This lab
rsmY-lux	pMS402 containing *rsmY* promoter region; Kn^r, Tmp^r	This lab
rsmZ-lux	pMS402 containing *rsmZ* promoter region; Kn^r, Tmp^r	This lab
pUT18C-*mtvA*	*mtvA* cloned into pUT18C for BTH assay; Cb^r	This study
pKT25- *mtvT*	*mtvT* cloned into pKT25 for BTH assay; Kn^r	This study
pUT18C-$mtvA^{1\text{-}50}$	$mtvA^{1\text{-}50}$ cloned into pUT18C for BTH assay; Cb^r	This study
pUT18C-$mtvA^{51\text{-}82}$	$mtvA^{51\text{-}82}$ cloned into pUT18C for BTH assay; Cb^r	This study
pUT18C-$mtvA^{L52A}$	$mtvA^{L52A}$ cloned into pUT18C for BTH assay; Cb^r	This study
pUT18C-$mtvA^{L55A}$	$mtvA^{L55A}$ cloned into pUT18C for BTH assay; Cb^r	This study
pUT18C-$mtvA^{Q58A}$	$mtvA^{Q58A}$ cloned into pUT18C for BTH assay; Cb^r	This study
pUT18C-$mtvA^{C59A}$	$mtvA^{C59A}$ cloned into pUT18C for BTH assay; Cb^r	This study
pUT18C-$mtvA^{D60A}$	$mtvA^{D60A}$ cloned into pUT18C for BTH assay; Cb^r	This study

pUT18C-*mtvA*^{W72A}	*mtvA*^{W72A} cloned into pUT18C for BTH assay; Cb^r	This study

Supplementary Table S2. Primers used in the study.

Primer	Sequence (5'→3')[a]	Application
pEX-*mtvA*-up-F	AATggtaccTTCACCTCCCCGCGG	For *mtvA* deletion
pEX-*mtvA*-up-R	AAAtctagaAACAGCTACGCCGGTAGGAAA	
pEX-*mtvA*-down-F	TTTtctagaTGAGAGTTCCCAATCGCACGG	
pEX-*mtvA*-down-R	ATAggacttTCTTCCCTGCCATTTGGCGA	
pEX-*mtvT*-up-F	AAAggtaccAACCGCTTATTGGAGAGG	For *mtvT* deletion
pEX-*mtvT*-up-R	TTAtctagaCCATAGATGAAGCCCTGCAGC	
pEX-*mtvT*-down-F	TTTtctagaGGTATTGTGATGCCCTGAGAG	
pEX-*mtvT*-down-R	TTAggatccACGCTGGATTTGAACAAT	
pEX-*mtvA*-up-F	AATggtaccTTCACCTCCCCGCGG	For *mtvTA* deletion
pEX-*mtvA*-up-R	AAAtctagaAACAGCTACGCCGGTAGGAAA	
pEX-*mtvT*-down-F	TTTtctagaGGTATTGTGATGCCCTGAGAG	
pEX-*mtvT*-down-R	TTAggatccACGCTGGATTTGAACAAT	
pEX-*dinJ*/*yafQ*-up-F	AATggattcTGGCTTTTAGGAGCTAAT	For *dinJ*/*yafQ* deletion
pEX-*dinJ*/*yafQ*-up-R	AATtctagaGATGTGCTCGTCGATACG	
pEX-*dinJ*/*yafQ*-down-F	ATTtctagaCCGTGTTGCAACTCGTG	
pEX-*dinJ*/*yafQ*-down-R	ATTaagcttTTCCGGGTACTTCTTGAT	
pEX-*glpT*-up-F	AAAggatccGCTTCGCCGAGCAGGTCA	For *glpT* deletion
pEX-*glpT*-up-R	AAAtctagaGGCATCGGGCAGTGGCGC	
pEX-*glpT*-down-F	AAAtctagaCTGACCCTGCGCAAACCC	
pEX-*glpT*-down-R	AAAaagcttGCATCCTCGATGGCGCCC	
mtvA -F	CGTTGCTGGCCGAACTCGGC	For *mtvA* mutant test
mtvA R	CTCCGGGGCCTCCAGGTCAC	
mtvTA-F	ATCaagcttTTGAAAACGACTATCCGT	For *mtvT* and *mtvTA* mutant test
mtvTA-R	TTTgaattcTCAGTCTTTCCCAACAAC	
dinJ/*yafQ*-F	AACgaattcATGTCTGCCAATGCTGTAGT	For *dinJ*/*yafQ* mutant test

dinJ/yafQ-R	AACaagcttTTACAGGCCAAGCTCGCT	
glpT-F	TGGAGGTTCGCTGAGCCA	For *glpT*mutant test
glpT-R	GTGGTCAGCGACCTCTGT	
mini-CTX-*mtvTA*-F	TTCtctagaCTGCTACTGTGGGTTTTAACT	For constructing *mtvTA* integrated plasmid
mini-CTX-*mtvTA*-R	ATCgaattcTCAGTCTTTCCCAACAACC	
pBBR1-*mtvA*-F	TCTgaattcTTGAAAACGACTATCCGTGC	For constructing pBBR1-*mtvA* and pET28a-*mtvA*
pBBR1-*mtvA*-R	ATCaagcttTCATGCTTCGTTTCCTACC	
pBBR1-*mtvT*-F	ATAgaattcATGAGCAAACGCCTGCAG	For constructing pBBR1-*mtvT* and pET28a-*mtvT*
pBBR1-*mtvT* -R	ATCaagcttTCAGTCTTTCCCAACAACC	
pMM-*mtvTA*-F	TCTggtaccTTGAAAACGACTATCCGTGC	For constructing pMMB67EH-*mtvTA*-Flag
pMM-*mtvTA*-R	GTCaagcttTCAGTCTTTCCCAACAACC	
pUT18C-*mtvA*-F	TCTtctagagTTGAAAACGACTATCCGTGC	For constructing pUT18C-*mtvA*
pUT18C-$mtvA^{1\text{-}50}$-R	AGTgaattcATATTCGGTGGCGACCTT	For constructing pUT18C-$mtvA^{1\text{-}50}$
pUT18C-$mtvA^{51\text{-}82}$-F	TTAtctagagATGGACCTCAGCACGCTA	For constructing pUT18C-$mtvA^{51\text{-}82}$
pUT18C-*mtvA*-R	ATCgaattcTCATGCTTCGTTTCCTACC	For constructing pUT18C-*mtvT*
pKT25-*mtvT*-F	TTAtctagagATGAGCAAACGCCTGCAG	For constructing pKT25-*mtvT*
pKT25-*mtvT*-R	AGTgaattcTCAGTCTTTCCCAACAACC	
pro-*mtvTA*-F	TTTctcgagCTGCTACTGTGGGTTTTA	Amplifying *mtvTA* promoter
pro-*mtvTA*-R	TTTggatccCGTTCTGTAGCCTCTTGC	
pro-P1-*mtvTA*-R	TTTggatccGTTCTCGGAGTGACATC	Amplifying $mtvTA_{-593\text{ to }-293}$ promoter
pro-P2-*mtvTA*-F	AGATCGAGTCTGCAGTCTG	Amplifying $mtvTA_{-350\text{ to }+1}$ promoter
pro-*dotA*-F	TTActcgagTGGTTTATGGCTCGGACTTTCGT	Amplifying *dotA* promoter
pro-*dotA*-R	TATggatccCCTCCGAGAAAGGCGGC	
pro-*dotD*-F	TTActcgagGGTCACAGGTGACCTATTTGG	Amplifying *dotD* promoter
pro-*dotD*-R	TATggatccCGTATCTGCTCGGTTGGT	

pro-*icmL*-F	TTActcgagTGATGGCGATCGCCG	Amplifying *icmL* promoter
pro-*icmL*-R	TATggatccACCGAACAAACCCCAACG	
pro-*icmP*-F	TTActcgagAAGTTTGGAATAACCAGA	Amplifying *icmP* promoter
pro-*icmP*-R	TATggatccTGCTGCCCCCGGACT	
pro-*lasI*-F	TGCTCTGATCTTTTCGGA	Amplifying *lasI* promoter
pro-*lasI*-R	GTACGATCATCTTCAC	
pro-*rsmY*-F	GCTGGGAAGGCTCGCGA	Amplifying *rsmY* promoter
pro-*rsmY*-R	CCTGCGCAATGTCCTGAC	
pro-*rsmZ*-F	CGGAAAACCTTAGACCCACT	Amplifying *rsmZ* promoter
pro-*rsmZ*-R	CAGGAGTGATATTAGCGATTCC	
pro-*exsA*-F	TTTCCGAATTGTACGGCC	Amplifying *exsA* promoter
pro-*exsA*-R	ACGCCCTCTTCCTTGTTT	
pro-*exsC*-F	GCCGTCTCCGCGCGGGA	Amplifying *exsC* promoter
pro-*exsC*-R	GGGGGCGCCTCCTAA	
dotD-F	TATACCGATGGCGCCGTTTAA	For the operon of *dotD*-*icmT*
icmT-R	GGCGGTATTGCGCCATTC	
icmP-F	ATGTCTGAAGTGAACAACAAGGG	For the operon of *icmP*-*icmO*
icmO-R	ATCAAACTAACAACGAGCTGAATG	
icmL-F	CTGGGGATCGCGCAATA	For the operon of *icmL*-*icmG*
icmG-R	AGCCGTTGGACATGATCA	
icmG-F	GTGCAACGCATCGATCCTGA	For the operon of *icmG*-*icmJ*
icmJ-R	TCAGGACACCCCTGCAAC	
icmJ-F	GTGCGTCCTTCAGTCGTCT	For the operon of *icmJ*-*icmB*
icmB-R	AACTGGTCGCCACTGGTCT	
pMM-*rsmA*-Flag-F	TTAgaattcATGCTGATTCTGACTC	Western blot
pMM-*rsmA*-Flag-R	AGTtctagaATGGTTTGGCTCTTG	
mtvA-N10A-F	ATCCGTGCGATTGGGGCCTCTCAGGGCAT CAC	For MtvA^{N10A} mutation
mtvA-N10A-R	GTGATGCCCTGAGAGGCCCCAATCGCACG	

	GAT	
mtvA-S11A-F	CGTGCGATTGGGAACGCTCAGGGCATCACAA	For MtvA^{S11A} mutation
mtvA-S11A-R	TTGTGATGCCCTGAGCGTTCCCAATCGCACG	
mtvA-T48A-F	GTGACCAAGGTCGCCGCCGAATATGACCTCA	For MtvA^{T48A} mutation
mtvA-T48A-R	TGAGGTCATATTCGGCGGCGACCTTGGTCAC	
mtvA-Y50A-F	AAGGTCGCCACCGAAGCTGACCTCAGCACGCT	For MtvA^{Y50A} mutation
mtvA-Y50A-R	AGCGTGCTGAGGTCAGCTTCGGTGGCGACCTT	
mtvA-L52A-F	GCCACCGAATATGACGCCAGCACGCTAGTCGC	For MtvA^{L52A} mutation
mtvA-L52A-R	GCGACTAGCGTGCTGGCGTCATATTCGGTGGC	
mtvA-L55A-F	TATGACCTCAGCACGGCAGTCGCCCAGTGTGA	For MtvA^{L55A} mutation
mtvA-L55A-R	TCACACTGGGCGACTGCCGTGCTGAGGTCATA	
mtvA-Q58A-F	AGCACGCTAGTCGCCGCGTGTGACCTGGAGGC	For MtvA^{Q58A} mutation
mtvA-Q58A-R	GCCTCCAGGTCACACGCGGCGACTAGCGTGCT	
mtvA-C59A-F	ACGCTAGTCGCCCAGGCTGACCTGGAGGCCCC	For MtvA^{C59A} mutation
mtvA-C59A-F	GGGGCCTCCAGGTCAGCCTGGGCGACTAGCGT	

mtvA-D60A-F	TAGTCGCCCAGTGTGCCCTGGAGGCCCCGGA	For MtvA^{D60A} mutation
mtvA-D60A-F	TCCGGGGCCTCCAGGGCACACTGGGCGACTA	
mtvA-W72A-F	CCGGACATCCAATGCGCGAGAACAGCTACGCC	For MtvA^{W72A} mutation
mtvA-W72A-F	GGCGTAGCTGTTCTCGCGCATTGGATGTCCGG	
qRT-*rpsL*-F	GTAAGGTATGCCGTGTACG	qRT-PCR of *rpsL*
qRT-*rpsL*-R	CACTACGCTGTGCTCTTG	
qRT-*dotA*-F	GCTGTCTCGACCGCTGAT	qRT-PCR of *dotA*
qRT-*dotA*-R	TGGATGCGACTGCCTGTAT	
qRT-*dotD*-F	TCGCTACGTTGTTGTCCG	qRT-PCR of *dotD*
qRT-*dotD*-R	ATGTTCTTCGCTGTTTCCTG	
qRT-*icmP*-F	GAGATATGCTCGTCAACAACG	qRT-PCR of *icmP*
qRT-*icmP*-R	ATTACCAACGCCCTTCCA	
qRT-*icmE*-F	CCGCCGTTATGAGTGCTG	qRT-PCR of *icmE*
qRT-*icmE*-R	GGTTTCAGTGACGCCAGTT	
qRT-*exsA*-F	AATCCTCTATGCCCATCAGTT	qRT-PCR of *exsA*
qRT-*exsA*-R	GCGATAGCTCTGGGTGAAA	
qRT-*exsC*-F	TCAACCGACTGCTTGCC	qRT-PCR of *exsC*
qRT-*exsC*-F	AACAGGGTGACGCCCAC	
qRT-*phzA1*-F	AACTGGACCACGGAAAGC	qRT-PCR of *phzA1*
qRT-*phzA1*-R	CACGTTGTGCCACTCCC	
qRT-*phzA2*-F	CAACTGGACCACGGAAAGC	qRT-PCR of *phzA2*
qRT-*phzA2*-R	TCTCGAAGATCCGCACGT	
qRT-*phzM*-F	CGATGTCGGTGCTCTGG	qRT-PCR of *phzM*
qRT-*phzM*-R	CGATCATGCGGGTTTCC	
qRT-*phzS*-F	CGACACCGCTGCGCCGGC	qRT-PCR of *phzS*

qRT-*phzS*-R	GGGTGCTTCCTTTTCTCG	
qRT-*lasR*-F	CCTTCATCGTCGGCAACT	qRT-PCR of *lasR*
qRT-*lasR*-R	TCGGCAGTACGCTCTGG	
qRT-*lasI*-F	AGATGATGATCCGTGCCG	qRT-PCR of *lasI*
qRT-*lasI*-R	GCCAGTCGCTGTTCCA	
qRT-*rhlR*-F	GTGGTGGGACGGTTTGC	qRT-PCR of *rhlR*
qRT-*rhlR*-R	GCCGGGTGAAGGGAAT	
qRT-*rhlI*-F	CTCTGAATCGCTGGAAGGG	qRT-PCR of *rhlI*
qRT-*rhlI*-R	GCGGATGGTCGAACTGG	
qRT-*pqsR*-F	GTTCTGCGATACGGTGAGC	qRT-PCR of *pqsR*
qRT-*pqsR*-R	CCTCGTCGATGGTGATGG	
qRT-*pqsA*-F	ACCCTGTTCGTCACCCTG	qRT-PCR of *pqsA*
qRT-*pqsA*-R	CTTGCCGTTGTCGTTGC	
qRT-*rsmA*-F	CGTGACGGTACTGGGTGT	qRT-PCR of *rsmA*
qRT-*rsmA*-R	CTTTCTGGATGCGCTGGT	
qRT-*rsmY*-F	CAGGACATTGCGCAGGA	qRT-PCR of *rsmY*
qRT-*rsmY*-R	TTTGGGCGGGGTTTTG	
qRT-*rsmZ*-F	TACAGGGAACACGCAACC	qRT-PCR of *rsmZ*
qRT-*rsmZ*-R	GTATTACCCCGCCCACTC	
qRT-*retS*-F	GACAACAGCATCTCCACCAA	qRT-PCR of *retS*
qRT-*retS*-R	GGCATCTCGCAGTCCATC	
qRT-*ladS*-F	GGACCGCCTTCCTTCTC	qRT-PCR of *ladS*
qRT-*ladS*-R	ACAGCAAGCCCACCTCC	
qRT-*gacA*-F	AACTGGCCCGCGAACT	qRT-PCR of *gacA*
qRT-*gacA*-R	GGTGACTACCACGACCTTGA	
qRT-*hcp1*-F	AAGACTCACGCCGAGGAAATC	qRT-PCR of *hcp1*
qRT-*hcp1*-R	GAGCAGGCCATCATCAGGTTG	
qRT-*hcp2*-F	GGCAACCCCCGCCTATATGT	qRT-PCR of *hcp2*
qRT-*hcp2*-R	GAAGCCCTGGACCATCACCT	

qRT-*hcp3*-F	CCTCAACGAGTATTGCTGCG	qRT-PCR of *hcp3*
qRT-*hcp3*-R	GGGTGTAGACGATGAAGGGC	

[a] Restriction sites are indicated in lowercase letters.

Supplementary Table S3. Antimicrobial susceptibility of *P. aeruginosa* strain PAD8(pPAD8) and PAD8(pPAD8$^{\Delta mtvTA}$).

**Strain**	**MIC (mg/L)**									
	**IPM**	**MEM**	**CAZ**	**PIP**	**FEP**	**CIP**	**LEV**	**TZP**	**ATM**	**CTX**
PAD8(pPAD8)	>8/R	>8/R	8/S	16/S	>16/R	>2/R	>8/R	16/S	>16/R	>32/R
PAD8(pPAD8$^{\Delta mtvTA}$)	>8/R	>8/R	2/S	8/S	8/S	>2/R	>8/R	8/S	>16/R	>32/R

ATM, aztreonam; CAZ, ceftazidime; CIP, ciprofloxacin; CTX, cefotaxime; FEP, cefepime; IPM, imipenem; LEV, levofloxacin; MEM, meropenem; PIP, piperacillin; TZP, piperacillin-tazobactam.

Supplementary Table S4. Plasmids identified by BLASTn search against pPAD8.

Plasmid	Species	Size (bp)	GC content	Coverage	Identity	Genbank accession
AR_0111 plasmid	*P. aeruginosa*	129422	58%	92%	99.99%	CP032256.1
PABCH42 plasmid	*P. aeruginosa*	129522	58%	92%	100.00%	CP056091.1
PA179 chromosome	*P. aeruginosa*	6580692	66%	92%	99.99%	CP058257.1
pF065	*P. aeruginosa*	138928	57%	88%	100%	CP115209.1
H05 plasmid	*P. aeruginosa*	143021	58%	89%	100.00%	CP093029.1
pNY7610-NR	*P. aeruginosa*	130277	58%	89%	98.86%	CP096915.1
pPYO_TB	*P. aeruginosa*	130306	58%	89%	98.88%	CP034355.1
plasmid unnamed1	*P. aeruginosa*	142661	57%	90%	99.92%	CP116726.1
pS04_90	*P. aeruginosa*	159187	58%	88%	98.83%	CP011370.1
plasmid unnamed1	*P. aeruginosa*	159128	58%	90%	99.76%	CP149855.1
RW109 plasmid 2	*P. aeruginosa*	151612	57%	88%	97.63%	LT969521.1
p2017-45-137A	*P. aeruginosa*	177464	57%	82%	99.57%	CP109686.1
pND6-2	*P． putida*	117003	58%	74%	93.83%	CP003589.1
plasmid unnamed1	*P. aeruginosa*	149191	57%	77%	93.64%	CP137941.1
pPA15W-NR	*P. aeruginosa*	116700	58%	74%	93.64%	MN961672.1
pLWPZF	*P． putida*	160969	58%	77%	93.63%	CP069081.1
p1_Teo6	*P. asiatica*	150161	58%	76%	93.64%	CP139777.1
pF045_1	*P. aeruginosa*	192539	57%	76%	93.63%	CP115240.1
p2	*Pseudomonas sp.*	173927	58%	77%	93.61%	CP146206.1
plasmid unnamed3	*P. juntendi*	132641	58%	62%	93,59%	CP125217.1
pNY11210-NR	*P. aeruginosa*	113722	58%	70%	89.60%	CP096959.1
p201330-IMP	*P. aeruginosa*	168687	58%	76%	93.74%	MN961671.1
pNY11382-NR	*Pseudomonas sp.*	148732	58%	77%	94.72%	CP097105.1
pF046	*P. aeruginosa*	130052	57%	76%	94.70%	CP115238.1
pS810a	*P. aeruginosa*	139453	59%	63%	83.47%	OL468819.1

Supplementary Table S5. The mRNA levels of T6SS genes in the mutant PAO1(pPAD8$^{\Delta mtvTA}$) versus the wild type strain PAO1(pPAD8) by RNA-seq data.

**Locus in PAO1**	**Gene name**	**Description**	**Fold changes (log2)**	***p*-value**
PA1512	*hcpA*	Secreted protein Hcp	-4.15321	5.80E-111
PA0263	*hcpC*	Secreted protein Hcp	-3.98792	2.63E-145
PA2368	*hsiF3*	HsiF3	-3.10183	9.19E-26
PA3486	*vgrG4b*	VgrG4b	-2.8247	1.28E-95
PA2366	*hsiC3*	HsiC3	-2.77204	3.00E-79
PA2369	*hsiG3*	HsiG3	-2.61492	6.41E-71
PA2367	*hcp3*	Hcp3	-2.60214	4.39E-59
PA5267	*hcpB*	secreted protein Hcp	-2.57879	9.25E-74
PA3294	*vgrG4a*	VgrG4a	-2.55542	1.02E-31
PA2365	*hsiB3*	HsiB3	-2.24361	4.11E-62
PA1665	*fha2*	Fha2	-2.14249	1.41E-58
PA2370	*hsiH3*	HsiH3	-2.13977	8.92E-36
PA1658	*hsiC2*	HsiC2	-2.13691	2.57E-74
PA5266	*vgrG6*	VgrG6	-2.12494	1.34E-46
PA1669	*icmF2*	IcmF2	-2.12031	3.67E-72
PA1661	*hsiH2*	HsiH2	-2.08295	6.56E-54
PA1664	*orfX*	OrfX	-2.02256	0.001474317
PA1657	*hsiB2*	HsiB2	-1.99858	4.62E-44
PA1667	*hsiJ2*	HsiJ2	-1.9890	1.86E-55
PA1663	*sfa2*	Sfa2	-1.95263	2.54E-55
PA1662	*clpV2*	clpV2	-1.93145	4.57E-59
PA1660	*hsiG2*	HsiG2	-1.923777017	3.11E-61
PA1666	*lip2*	Lip2	-1.88752	2.18E-40
PA1668	*dotU2*	DotU2	-1.84524	2.48E-36
PA2362	*dotU3*	DotU3	-1.84205	1.52E-17

PA2371	*clpV3*	ClpV3	-1.83981	4.53E-34
PA1659	*hsiF2*	HsiF2	-1.78447	2.05E-35
PA1670	*stp1*	Stp1	-1.74099	1.14E-29
PA1511	*vgrG2a*	VgrG2a	-1.72571	1.76E-42
PA5090	*vgrG5*	VgrG5	-1.69077	3.38E-35
PA2361	*icmF3*	IcmF3	-1.50997	6.35E-30
PA0262	*vgrG2b*	VgrG2b	-1.49249	1.10E-39
PA1656	*hsiA2*	HsiA2	-1.45756	1.91E-46
PA2360	*hsiA3*	hypothetical protein	-1.41876	4.10E-23
PA2363	*hsiJ3*	HsiJ3	-1.28859	1.31E-22
PA0085	*hcp1*	Hcp1	-1.2854	3.67E-19
PA0088	*tssF1*	TssF1	-1.19853	2.17E-11
