# Supplementary material for: A plasmid-encoded inactive toxin–antitoxin system MtvT/MtvA regulates plasmid conjugative transfer and bacterial virulence in Pseudomonas aeruginosa
Source: Nucleic Acids Res. 2025 Feb 14;53(4):gkaf075. doi: 10.1093/nar/gkaf075 (PMC11826091; doi:10.1093/nar/gkaf075)
Supplement: gkaf075_Supplemental_Files [file gkaf075_supplemental_files.zip › Supplementary Figure.pdf]

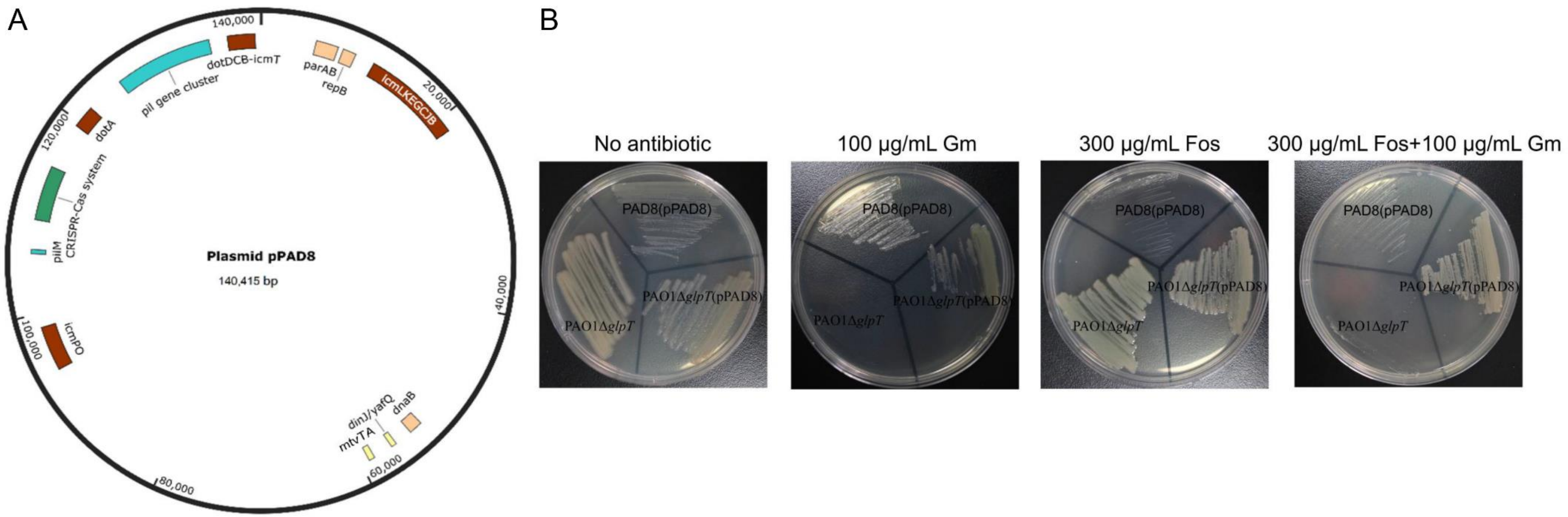

Supplementary Figure S1. (**A**) Circular map of plasmid pPAD8, the position of the *dot*/*icm* system genes (brown), the *pil* gene cluster (cyan), putative toxin-antitoxin genes (yellow), CRISPR-Cas system genes (green), and plasmid replication and partition genes (light orange) are indicated in the plasmid. (**B**) Growth of the donor PAD8(pPAD8), the recipient PAO1Δ*glpT*, and the transconjugant PAO1Δ*glpT*(pPAD8) were detected under different antibiotics. Gm: gentamicin, Fos: fosfomycin.

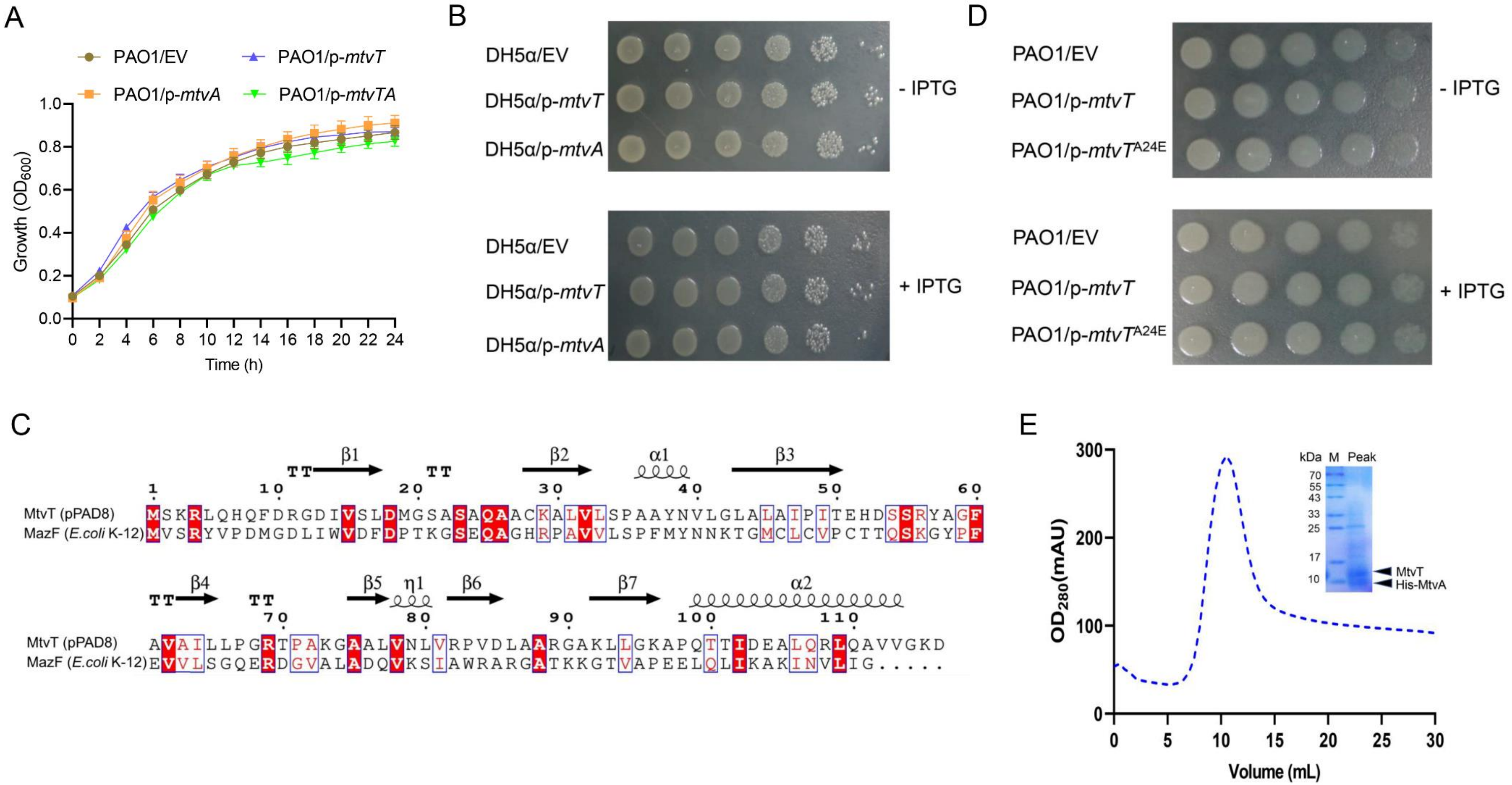

Supplementary Figure S2. (**A**) Growth of the strain PAO1 upon overexpressing *mtvA*, *mtvT*, and *mtvTA* were determined using a Synergy 2 Microplate Reader (BioTek). Three independent cultures of each strain were tested, and error bars indicate the standard error of the mean (n = 3).

(**B**) Growth of *E. coli* strain DH5α harboring a vector expressing the *mtvT* or *mtvA* on LB agar with or without 0.5 mM IPTG at 37°C. EV represents empty vector pBBR1MCS-5. Overnight cultures were spotted on LB medium plates by successive ten-fold dilution. (**C**) Multiple sequence alignment constructed by ClustalW to compare the amino acid sequence identity of MtvT and MazF (*E. coli* K-12). Conserved amino acid are highlighted in red. (**D**) Growth of the strain PAO1 harboring a vector expressing the *mtvT* or *mtvT*^{A24E} on LB agar with or without 0.5 mM IPTG at 37°C. EV represents empty vector pBBR1MCS-5. Overnight cultures were spotted on LB medium plates by successive ten-fold dilution. (**E**) MtvT and MtvA form a complex *in vitro*. His-tagged MtvA and untagged MtvT were coproduced via pET28a-His-*mtvA*-*mtvT*.

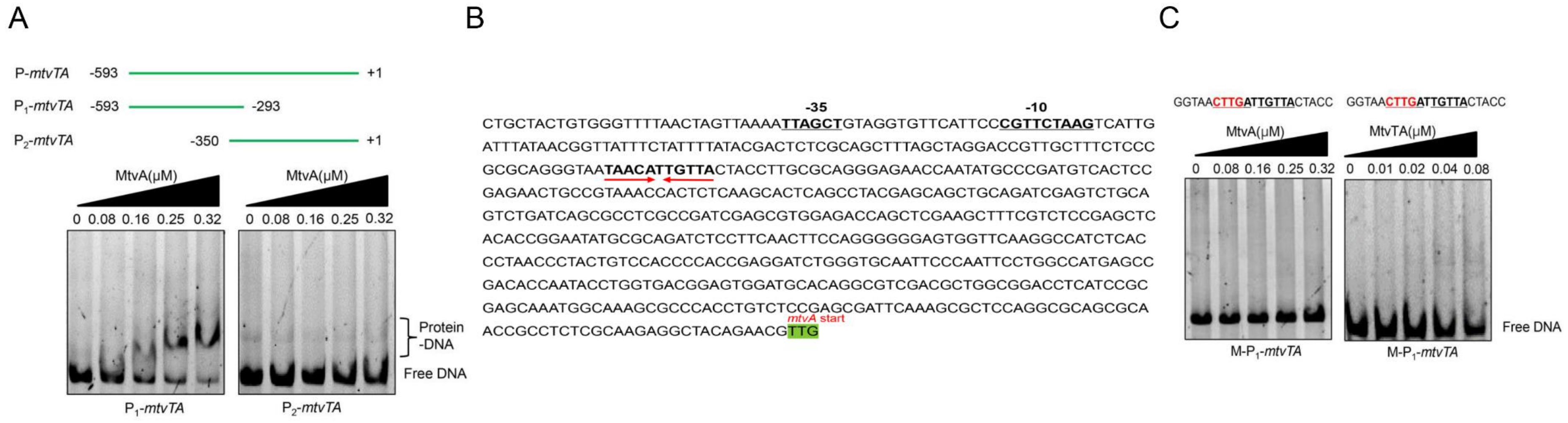

Supplementary Figure S3. (**A**) EMSA showed that MtvA bind and shift the promoter of P-*mtvTA*. The positions of DNA probe are indicated in the upper panel. Numbers represent the position relative to the *mtvA* start codon. MtvA bind and shift P_1-*mtvTA* but not P_2-*mtvTA*. (**B**) Nucleotide sequence of the promotor region *mtvTA*. The −10 and −35 regions are marked in bold letters. The palindromic sequence is shown by the red arrow and bold letters. Start codon of *mtvA* is highlighted in green. (**C**) EMSA showed that MtvA and the MtvTA complex not bind and shift the mutant promoter M-P_1-*mtvTA*. The binding of MtvA and the complex MtvtA to M-P_1-*mtvTA* was abolished after random mutating the palindrome to5′-TAACATTGTTA-3′.

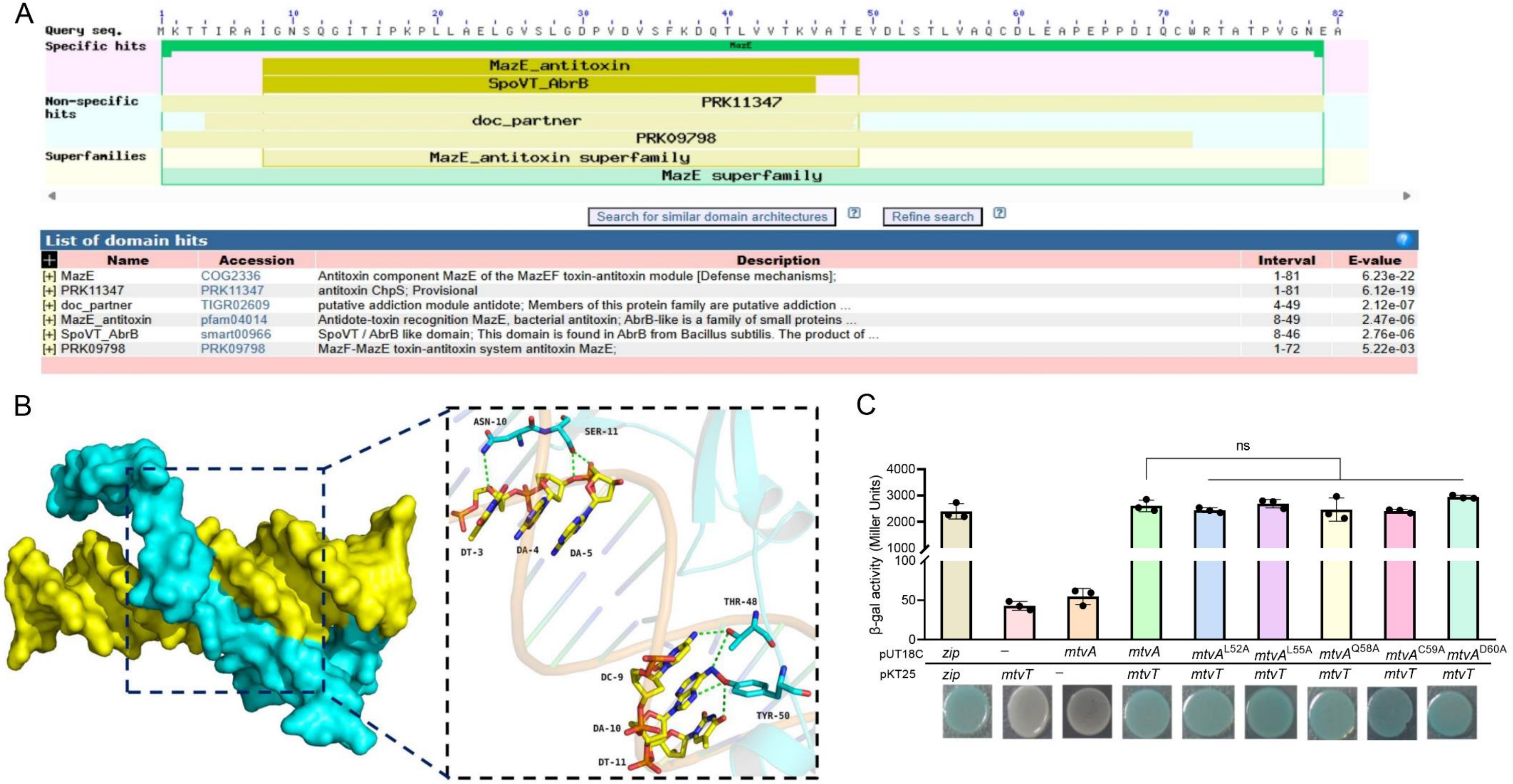

Supplementary Figure S4. (**A**) Predicted structure domain of protein MtvA by the Conserved Domain Database from NCBI. (**B**) Molecular

docking model between MtvA and palindromic sequence 5′-GGTAATAACATTGTTACTACC-3′ of the promoter region P-*mtvTA* with overall and enlarged view. (**C**) The BACTH assay were performed to assess interactions between protein MtvA, $MtvA^{L52A}$, $MtvA^{L55A}$, $MtvA^{Q58A}$, $MtvA^{C59A}$ as well as $MtvA^{D60A}$ and MtvT. The quantified data from different experiments are presented as mean ± s.d. of three biological replicates. The *P* values were calculated by two-tailed Student's *t* test. ns: not significant.

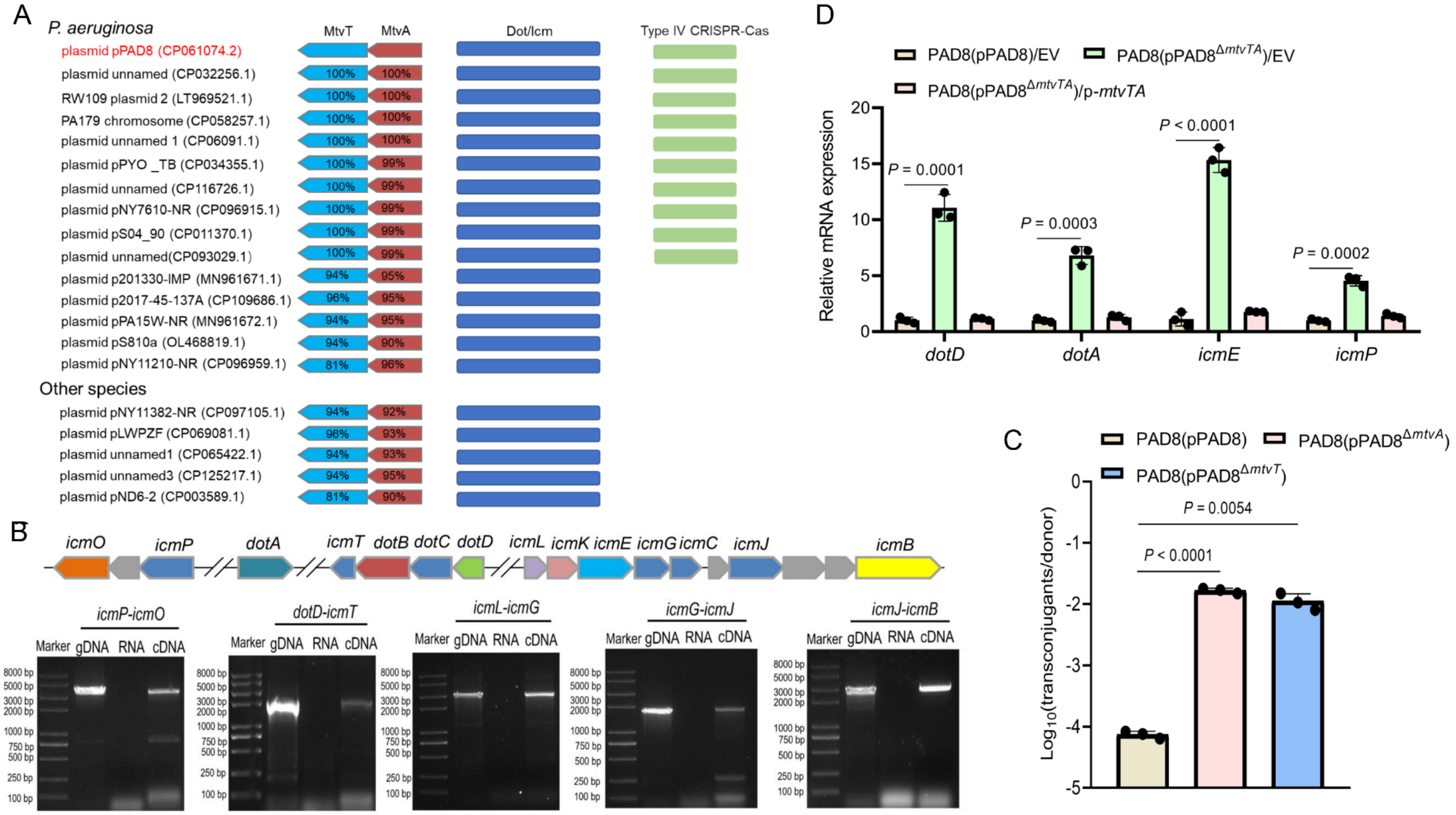

Supplementary Figure S5. (**A**) The distribute of the *mtvTA* system, *dot/icm*, and type IV CRISPR-Cas system in the Inc_{pPAD8} family plasmids,

MtvT, MtvA, *dot*/*icm* system, and type IV CRISPR-Cas system were shown by dark blue, brown, blue, and green, respectively. (**B**) The *dot*/*icm* system genes are distributed in four centralized locations in the plasmid pPAD8, different functional genes are indicated in the different colour, unknown functional genes are shown in gray (Top). 1% agarose gel loaded with the amplified fragments of *icmP-icmO*, *dotD-icmT*, *icmL-icmG*, *icmG- icmJ*, and *icmJ-icmB* (Bottom). The respective primers were used for reverse transcription. Genomic DNA (gDNA) and RNA were used as positive and negative controls, respectively. (**C**) Conjugative transfer efficiency of the wild-type PAD8(pPAD8), the single mutant PAD8(pPAD8$^{\Delta mtvA}$), and single the mutant PAD8(pPAD8$^{\Delta mtvA}$) were detected. Conjugation assays conducted on solid plates. EV represents empty vector pMMB67EH. (**D**) The relative mRNA levels of *dotD*, *dotA*, *icmE*, and *icmP* were determined in the wild-type PAD8(pPAD8), the mutant PAD8(pPAD8$^{\Delta mtvTA}$), and its complemented strain by qRT-PCR. EV represents empty vector pMMB67EH. For **C** and **D**, the quantified data from different experiments are presented as mean ± s.d. of three biological replicates. The *P* values were calculated by two-tailed Student’s *t* test.

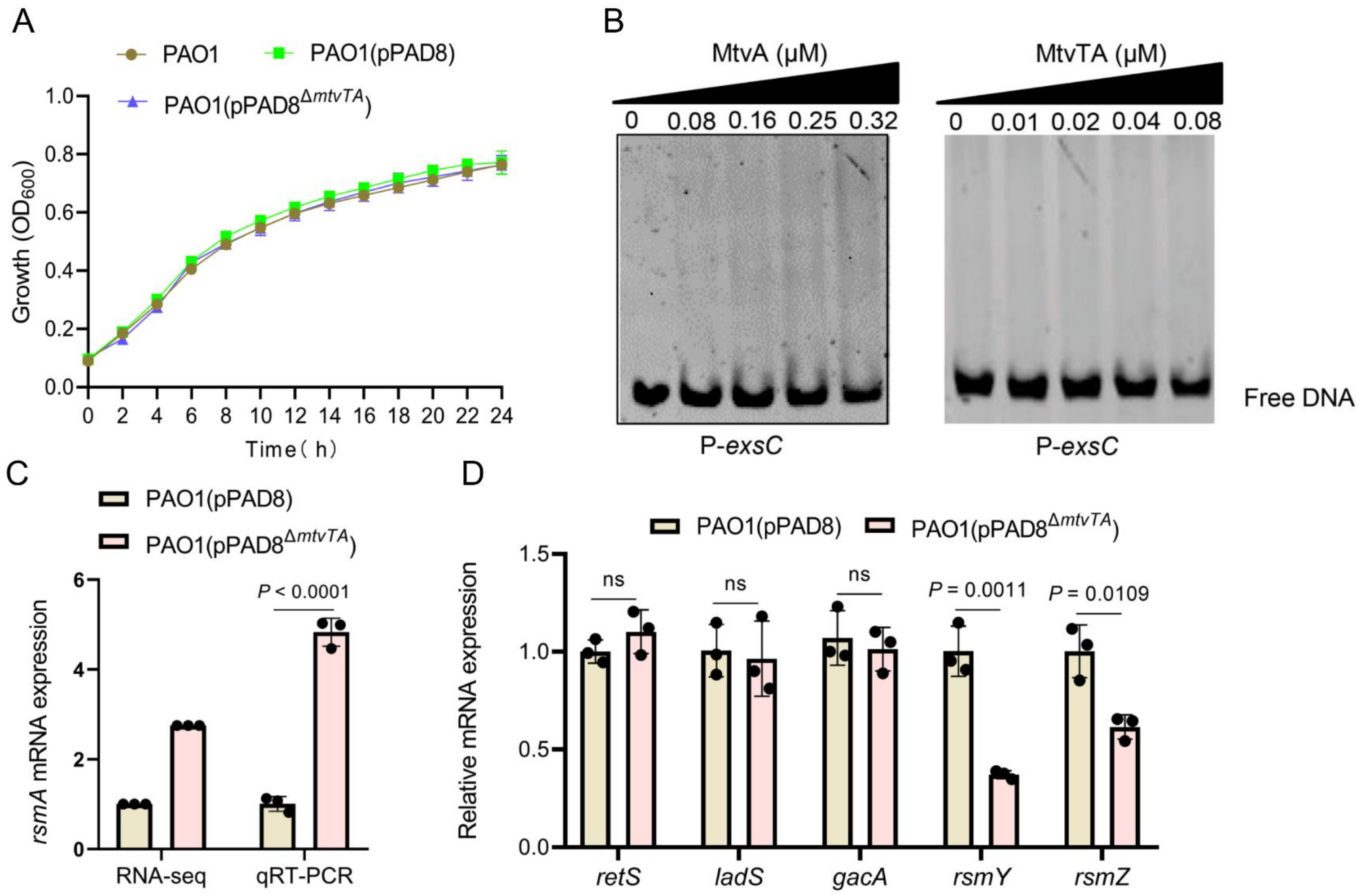

Supplementary Figure S6. (**A**) Growth of the wild-type PAO1, the wild-type PAO1(pPAD8), and the mutant PAO1(pPAD8$^{\Delta mtvTA}$) were

determined by a Synergy 2 Microplate Reader (BioTek). Three independent cultures of each strain were tested, and error bars indicate the standard error of the mean (*n* = 3). (**B**) EMSA showed that MtvA and the MtvTA complex not bind and shift the promoter P-*exsC*. Each reaction mixture contains 1.0 ng/mL of PCR products of P-*exsC*. The protein concentrations are indicated above the lane. Data representative of *n* = 3 biologically independent experiments. (**C**) Fold changes of the mRNA levels of the gene *rsmA* in the wild-type PAO1(pPAD8) and the mutant PAO1(pPAD8$^{\Delta mtvTA}$) from RNA-seq data and qRT-PCR data, respectively. (**D**) The relative mRNA levels of *retS*, *ladS*, *gacA*, *rsmY*, and *rsmZ* were determined in the wild type PAO1(pPAD8) and the mutant PAO1(pPAD8$^{\Delta mtvTA}$) by qRT-PCR. For **C** and **D**, the quantified data from different experiments are presented as mean ± s.d. of three biological replicates. The *P* values were calculated by two-tailed Student's *t* test.

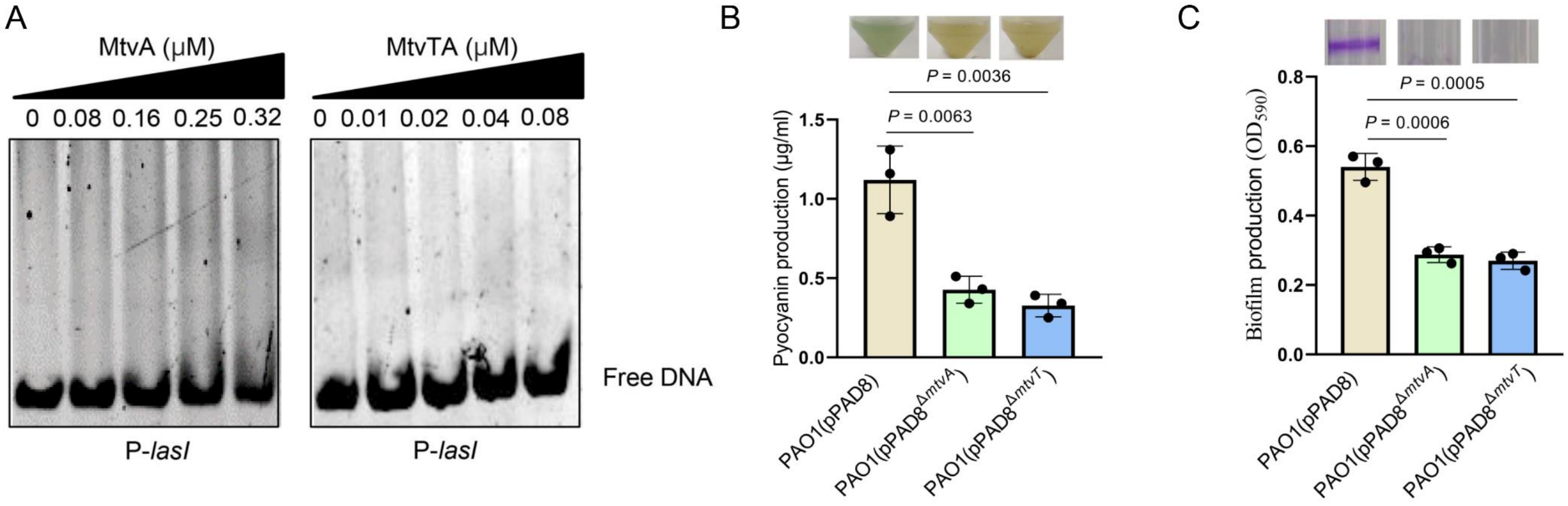

Supplementary Figure S7. (**A**) EMSA showed that both MtvA and the MtvTA complex not bind and shift the promoter P-*lasI*. Each reaction mixture contains 1.0 ng/mL of PCR products of P-*lasI*. The protein concentrations are indicated above the lane. Data representative of $n = 3$ biologically independent experiments. (**B**) The pyocyanin production of the wild-type PAO1(pPAD8), the single mutant PAO1(pPAD8$^{\Delta mtvA}$), and the single mutant PAO1(pPAD8$^{\Delta mtvT}$) were detected after culture in LB medium for 12 h. (**C**) The biofilm formation of the wild-type PAO1(pPAD8), the single mutant PAO1(pPAD8$^{\Delta mtvA}$), and the single mutant PAO1(pPAD8$^{\Delta mtvT}$) were displayed with crystal violet staining (Top) and quantified with optical density measurement (Bottom).
